# Analysis of Spo0M function in *Bacillus subtilis*

**DOI:** 10.1371/journal.pone.0172737

**Published:** 2017-02-24

**Authors:** Luz Adriana Vega-Cabrera, Adán Guerrero, José Luis Rodríguez-Mejía, María Luisa Tabche, Christopher D. Wood, Rosa-María Gutiérrez-Rios, Enrique Merino, Liliana Pardo-López

**Affiliations:** 1 Instituto de Biotecnología, Universidad Nacional Autónoma de México, Apdo, Cuernavaca, Morelos, México; 2 Laboratorio Nacional de Microscopía Avanzada, Avenida Universidad 2001, Universidad Autónoma del Estado de Morelos, Cuernavaca, Morelos, México; Universitat Pompeu Fabra, SPAIN

## Abstract

Spo0M has been previously reported as a regulator of sporulation in *Bacillus subtilis*; however, little is known about the mechanisms through which it participates in sporulation, and there is no information to date that relates this protein to other processes in the bacterium. In this work we present evidence from proteomic, protein-protein interaction, morphological, subcellular localization microscopy and bioinformatics studies which indicate that Spo0M function is not necessarily restricted to sporulation, and point towards its involvement in other stages of the vegetative life cycle. In the current study, we provide evidence that Spo0M interacts with cytoskeletal proteins involved in cell division, which suggest a function additional to that previously described in sporulation. Spo0M expression is not restricted to the transition phase or sporulation; rather, its expression begins during the early stages of growth and Spo0M localization in *B*. *subtilis* depends on the bacterial life cycle and could be related to an additional proposed function. This is supported by our discovery of homologs in a broad distribution of bacterial genera, even in non-sporulating species. Our work paves the way for re-evaluation of the role of Spo0M in bacterial cell.

## Introduction

*Bacillus subtilis*, an aerobic Gram-positive bacterium, has been widely used as a model to study processes such as sporulation and heterologous protein production. Sporulation in *B*. *subtilis* is a cell differentiation process, which allows cells to withstand adverse conditions. Sporulation in *B*. *subtilis* is triggered by the phosphorylation of the sensor histidine kinases KinA-E that in turn initiates a phosphorelay pathway which culminates in the phosphorylation of the master regulator of sporulation, Spo0A [1]. The phosphorylation level of Spo0A determines the alternative differentiation states that the cell adopts in response to adverse environmental circumstances [1]. These states include competence, biofilm formation, cannibalism and sporulation, with the latter representing the last resort for cell survival.

The first stage of differentiation is known as stage zero and encompasses the decision of the cell to enter the process. During stage I the bacterial chromosome is duplicated and an axial filament is formed, with the chromosome bound to both cell poles. At stage II, cells acquire sporulation commitment and are subject to asymmetrical division, an irreversible decision that generates the forespore and the mother cell. In stage III the mother cell membrane migrates around the forespore, in a process known as engulfment. Stage IV and V include the formation of the protective structures of the spore, the coat and the cortex. In stage VI and stage VII the spore matures and is released after the lysis of the mother cell [2]. From the beginning of the asymmetrical division both new compartments turn on different genetic programs directed by specific sigma factors that drive the subsequent steps.

The Spo0M protein has been shown to be involved in sporulation [3]; however, the mechanism by which it performs this function has not been elucidated. A *spo0M* null mutant is viable; however, when the intracellular concentration of Spo0M is low, sporulation is diminished and blocked at stage zero; additionally, cells at the vegetative stage are susceptible to lysis [3]. Similarly, overexpression of Spo0M also inhibits sporulation at stage zero, suggesting that Spo0M concentrations are strictly regulated. This regulation is known to be mediated through proteolytic degradation by the metalloprotease FtsH, that has been shown to degrade Spo0M *in vitro* [4]. It was first reported that Spo0M is under the control of the stationary phase sigma factor σ^H^ [3], however, recent work has indicated that Spo0M is also regulated through the stress responsive sigma factor σ^W^ [5–7]. Finally, it has been reported that when intracellular concentrations of Spo0M are low, the expression of Spo0A is down-regulated [3]. To date, the mechanisms by which Spo0M regulates sporulation, the details of Spo0M’s association with Spo0A, and the involvement of Spo0M in processes other than sporulation have not been reported.

Cell division and sporulation require the assembly of a septum to initiate the cascade of events that will ultimately produce a new cell: a daughter cell in the case of cell division and a spore in the case of sporulation. Assembly of the Z ring, a structure composed of FtsZ subunits, is the first step in both processes, and most of the elements involved in regulating the polymerization and membrane tethering of FtsZ are also shared between the two. The Z ring is stabilized and tethered to the cell membrane through positive regulators such as FtsA, [8], ZapA [9] and SepF [10] and by negative regulators like EzrA [11]. Medial and asymmetrical divisions differentiate in the way the chromosome is segregated and how cytokinesis is achieved after septum formation. During medial division, the MinCD system avoids the formation of aberrant septa near the cell poles [12,13] and the Nucleoid Occlusion (NOC) system prevents septum formation in the middle of the cell before the chromosome is segregated [12,13]; cytokinesis is achieved under the direction of a large number of elements, including transporters (e.g., FtsE/X) [14], cell wall synthesis machinery (e.g., PBP1A and PBP2) [15], and various proteins that act to recruit additional components(reviewed by [16–18]). The protein complex involved in this process is known as the divisome.

During sporulation, the SpoIIE phosphatase regulates the re-localization of the septum from the medial to polar position [19]; after septum establishment one third of the chromosome is retained in the forespore and the rest of the chromosome is translocated by the SpoIIIE ATPase [20]. Unlike medial division, cell wall is not newly synthesized but removed, allowing engulfment to proceed [21] and cytokinesis is replaced by the lysis of the mother cell.

In the current study, we provide evidence that Spo0M functions are not restricted to sporulation, but could also regulate vegetative stage processes of *B*. *subtilis*, specifically, during cell division. We found that the *spo0M* null mutant is morphologically altered, showing elongation and membrane defects not present in wild type cells. Here we demonstrate that Spo0M expression begins during early stages of growth, which suggest an additional role in the vegetative stage of life of *B*. *subtilis*. We analyzed the Spo0M localization within the cells using a fluorescent fusion with the DsRed protein, finding that this fusion is located to the cell poles and the septum region, where it colocalizes with the cell division related protein ZapA. Finally, we determined that Spo0M interacts with cell division and membrane organization proteins, chaperones, kinases, proteases and proteins involved in sporulation; this supports our hypothesis that Spo0M is not restricted to the sporulation stage and instead it is involved in other processes during the vegetative growth in *B*. *subtilis*. Our findings lay a foundation for future investigation of the unexplored functions of Spo0M, whose role has been previously reported only as a regulator of sporulation.

## Materials and methods

### General methods

The strains we used in this work are isogenic derivatives of *B*. *subtilis* 168 [22] or PY79 [23]; additionally, we used the hypercompetent strain SCK6 [24]. Competent *B*. *subtilis* cells were prepared as previously described [25]. Strain FG347, *amyE*::P_*xyl*_-*gfp*-*zapA* (*cat*) [9] was previously constructed and kindly provided by Dr. Sarah Wacker from Dr. R. Losick’s group. Strain Δ0M was constructed by allelic replacement of the *spo0M* open reading frame with a kanamycin resistance cassette, constructed using a long-flanking PCR method [26]. This construct was incorporated in the SCK6 and FG347 strains to generate *spo0M* null mutants in these genetic backgrounds. The Spo0M-DsRed fusion construct was generated by designing a pSGGS plasmid (S2 Table) to possess the same genetic backgrounds mentioned above. Antibiotics were used at the following concentrations: chloramphenicol, 5 μg mL^-1^; erythromycin, 1 μg mL^-1^; kanamycin, 15 or 30 μg mL^-1^; spectinomycin, 100 μg mL^-1^. DNA manipulation and cloning were performed according to standard methods [27]. *Taq* and *Phusion* DNA polymerases (Thermo Scientific, Waltham, MA, USA) and an Expand Long Template PCR system (Roche, Mannheim, Germany) were used for PCR reactions. The oligonucleotide primers used in this study are listed in S3 Table. Oligonucleotide synthesis and sequencing was performed by the Departmental DNA Synthesis and Sequencing facility of IBt-UNAM. The strains and plasmids used in this study are listed in S2 Table. Plasmid construction is further described in the S1 File.

### Spore resistance test

Strains were cultured in 2XSG sporulation agar for 6 days at 37°C. After this, viable spores were collected and quantified. A total of 1x10^6^ spores were used for analysis. To assay wet heat resistance, spores were diluted in 1 mL of sterile water and incubated at 80°C for 30 min. To assay chemical resistance, spores were diluted in 1 mL of sterile water, and 1500 pm of commercial bleach was added. The spores were then incubated with the bleach for 30 min, harvested and resuspended in sterile water. After each treatment, a 100 μL aliquot of sample was plated on LB agar, and the number of viable spores was quantified. We performed at least three biological replicates of each experiment mentioned above.

### Microscopy

Cultures were grown at 37°C in Luria-Bertani (LB) medium [28], Spizizen minimal medium [29] and 2XSG sporulation medium [30]. The last two were supplemented with 0.1% glucose and 0.1% bacto casamino acid. If necessary, xylose was added (0.1% final concentration) to induce the expression of GFP:ZapA. Cells were harvested, resuspended in phosphate-buffered saline (PBS), and fixed with 4% paraformaldehyde (PFA), if necessary. The cells were stained with 0.01 mg mL^-1^ FM4-64 (F34653, Life Technologies, Carlsbad, CA, USA) (final concentration), 0.01 mg mL^-1^ Hoechst 33342 (H3570, Invitrogen, Carlsbad, CA, USA) or 0.01 mg mL^-1^ 2-(4-amidinophenyl)-1H-indole-6-carboxamidine (DAPI) (D1306, Invitrogen) (final concentration). Following this, the cells were observed with an inverted Olympus FV1000 IX81 confocal microscope using a UPLSAPO 60XS/1.3 Ph3 silicon oil immersion objective; or with an Olympus IX81 TIRF microscope using a UAPON-OTIRF 100X/1.49 oil immersion objective. Fluorescence measurements were always performed with the same hardware settings (e.g., laser intensity, sampling, acquisition rate, pinhole and amplification settings) in the Laboratorio Nacional de Microscopía Avanzada (National Laboratory of Advance Microscopy). Images were analyzed with open-source Fiji—ImageJ software [31].

### Super-resolution microscopy

All super-resolution imaging measurements were performed on an Olympus IX-81 inverted microscope configured for total internal reflection fluorescence (TIRF) excitation (cellTIRF Illuminator; Olympus). The excitation angle was set up such as the evanescence field had a penetration depth of about ~500 nm (Xcellence software v1.2; Olympus Soft Imaging Solution GMBH). The samples were continuously illuminated using excitation sources depending on the fluorophore used. Blue (Hoechst), green (GFP) and red (FM4-64; Ds-RED) were excited with either a 405, 491-nm or a 568-nm diode-pumped solid-state laser, respectively. The maximum laser power, measured at the back of the focal plane of the objective lens, ranged between 20 to 25 mW, depending in the laser line used. Beam selection and modulation of laser intensities were controlled via Xcellence software v.1.2. A full multiband laser cube set was used to discriminate the selected light sources (LF 405/488/561/635 A-OMF, Bright Line; Semrock). Fluorescence was collected using an Olympus UApo N 100×/1.49-numerical-aperture oil-immersion objective lens with an extra 1.6× intermediate magnification lens. All movies were recorded on a full chip mode of an EMCCD camera (Andox, Ixon 888) at 100 nm per pixel. Sub-diffraction images were derived from the Super Resolution Radial Fluctuation (SRRF) analysis [32]. For each super-resolution reconstruction (x, y, z), five to then serial stacks were acquired within the evanescent field, with an axial ‘z’ spacing of 100 nm. Each serial stack, composed of 300 temporal images collected with an exposure time of 5–20 ms and at 6–7 Hz, was fed into NanoJ-core and NanoJ-SRRF plugins of Image J [31,32]. The following parameters were considered: ring radius 0.5, radiality magnification 10, axes in ring 10; all other parameters were set up as the default options. The radiality maps were drift corrected using pre-calculated drift tables obtained with the Estimate Drift tool of NanoJ-SRRF, considering a time averaging of 300 images. These drift-corrected radiality maps were then finally integrated on a super-resolution image by means of calculating the second order cumulant of the temporal radiality auto-correlations. Fluorescence measurements were always performed with the same hardware settings (e.g., laser intensity, sampling, acquisition rate, pinhole and amplification) in the Laboratorio Nacional de Microscopía Avanzada (National Laboratory of Advance Microscopy). Images were analyzed with Fiji—ImageJ software [31].

### Imaging flow cytometry

Septum formation was analyzed by quantifying fluorescence intensity in an Amnis Image Stream Mark II imaging flow cytometer. Cells from the strains FG347 and GFP:ZapA:Δ0M were grown in rich medium until exponential phase (OD_600_ 0.6–0.8) and GFP:ZapA expression was induced by adding 0.1% xylose (final concentration) for 4h. Samples were run until 5x10^3^ cells were recorded for each biological repetition and the reported data correspond to three biological repetitions.

### Western blot

SDS-PAGE was performed as reported by Laemmli [33]. Primary antibodies were detected using HRP-conjugated secondary antibodies, which was followed by enhanced chemiluminescence detection (ECL; GE, Amersham Biosciences, Little Chalfont, Buckinghamshire, UK) according to the manufacturer’s protocol. The same procedure was followed to evaluate co-immunoprecipitation assays using FtsZ-FLAG and GFP-FLAG. The following commercial antibodies were acquired: anti-FtsZ (AP10076PU-N, Agrisera, Vänäs, Sweden), anti-GFP (GTX113617, GeneTex, Alton Pkwy, Irvine, CA, U.S.), anti-DsRed (GTX59862, GeneTex), anti-DsRed (GTX82561, GeneTex) and anti-FLAG (F1804, Sigma).

### Interactome

T7-MAT-Spo0M-FLAG plasmid construction is described in S1 File. The plasmid was transformed into the BL21-DE3 *E*. *coli* strain, and protein expression was induced by incubation with 0.5 mM IPTG (final concentration) for 6–8 h in LB medium. After induction, the cells were harvested and resuspended in lysis buffer (50 mM HEPES, pH 7.4; 2.5 mM EDTA; 150 mM NaCl; 10% glycerol; 1% NP40; protease inhibitor cocktail (Roche); phosphatase inhibitor cocktail (Roche)). The samples were sonicated, and total protein extract was collected. Total protein extract from an empty vector (pT7-MAT-FLAG) was used as a control. Total protein extracts were also obtained from the Bs1A1 wild type *B*. *subtilis* strain, as described above, and were mixed with the control and experimental samples for 2 h at 4°C. Following this, both mixtures were incubated with anti-FLAG agarose beads (Sigma-Aldrich, St. Louis, MO, USA) for 2 h at 4°C. The samples were then washed with lysis buffer and loaded onto an SDS-PAGE gel. The gel was stained with Coomassie Brilliant Blue R-250 Staining Solution (BioRad, Hercules, CA, USA) and analyzed to identify differential bands between the control and experimental samples. We performed three biological replicates of the experiment. Bands of interest were excised and sent to the Proteomics Discovery Platform in the Institute de Recherches Cliniques de Montreál, Canada, for mass spectrometry analysis. The results were analyzed using Scaffold 4.4.7 commercial software [34].

### Coimmunoprecipitation assays

Immunoprecipitations with the anti-FtsZ or anti-GFP antibodies were performed by mixing 1 mg of total protein from the lysates of the Spo0M:DsRed or the induced GFP:ZapA/Spo0M:DsRed strains with an anti-DsRed antibody for 3 h at 4°C. After this, 50 μL protein A agarose beads (sc-2001, Santa Cruz Biotechnology, Dallas, Texas, U.S.) were added to the mixture and incubated for 3 h at 4°C. The presence of FtsZ or GFP:ZapA was revealed by Western blotting with an anti-FtsZ or anti-GFP antibody. In the reverse order experiment, 1 mg of total protein from lysate of the Spo0M:DsRed or strain was mixed with an anti-FtsZ antibody for 3 h at 4°C. After this, 50 μL protein A agarose beads were added to the mixture and incubated for 3 h at 4°C. The presence of Spo0M:DsRed was revealed by Western blotting with an anti-DsRed antibody.

### Bioinformatics procedures

#### Analysis of the sporulation phenotypes of strains carrying the *spo0M* gene

A list of representative bacteria carrying the *spo0M* gene was obtained from the KEGG database [35]. Each member of this set of organisms was classified as sporulating or non-sporulating bacteria in accordance with the BacMap database [36] or based on the presence of gene markers associated with the sporulation process in the genera *Bacillus* [37] and *Streptomyces* [38,39]. The results of the analysis were represented as a phylogenetic tree. To construct this tree, a multiple alignment of the concatenation of 31 ortholog protein sequences that were selected by Ciccarelli *et al*. [40] for being universally present among sequenced genomes was obtained using the MUSCLE alignment program [41]. This sequence alignment was used to estimate genetic distances using PROTDIST from J. Felsenstein’s PHYLIP phylogeny inference package program [42]. On the basis of these estimated distances, successive clustering of lineages was performed using a neighbor-joining algorithm reported by Zhang and Nei [43], as implemented in the NEIGHBOR program [43]. The resulting tree was drawn using the iTol web server [44].

#### Analysis of σ^A^ promoter sequences of *spo0M* gene

The transcription units of representative organisms of the genus *Bacillus* carrying the *spo0M* gene were obtained from the ProOpDB operon database [45] and their corresponding 5´ intergenic regions were obtained from the GeConT web server [46]. These sequences were used to search for potential binding sites of the housekeeping σ^A^ using the algorithm of Mulligan *et al*. [47] and then represented as a logo using the WebLogo server [48].

## Results and discussion

### Spo0M homologs are found in non-sporulating bacteria

**Fig 1:**
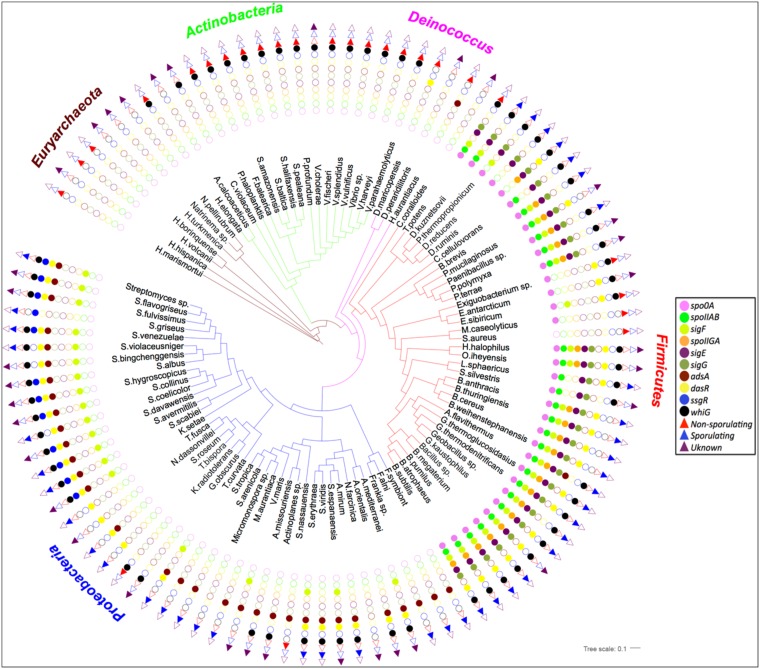
Spo0M homologs are found in non-sporulating bacteria. A list of representative bacteria carrying the *spo0M* gene was obtained, and each member was classified as sporulating (blue triangle) or non-sporulating (red inverted triangle) or not determined (purple triangle) according to the BacMap database [36] or based on the presence of gene markers associated with the sporulation process (color circles). A multiple alignment of the concatenation of 31 orthologous protein sequences was obtained and used to estimate the genetic distances of the organisms. A clustering of lineages was performed and used to construct the shown phylogenetic tree.

We started with the analysis of the function of Spo0M by determining which bacteria have homologs of this protein. As described in Materials and methods, we identified orthologs to Spo0M by employing the KEGG orthologous database. All the prokaryote proteins considered in the set were related to the KO K06377 data set, defined as sporulation-control protein, using the default parameters [35]. Then, we classified the bacteria in the set as sporulating, non-sporulating or not determined, considering the annotations provided by the BacMap database [36]. Using the same approach as for Spo0M, we searched for orthologs of different proteins described to participate in sporulation, such Spo0A, the master regulator of sporulation, the sigma factors known to induce the genes involved in the cascade of sporulation both in *Bacilli* and *Streptomyces* (see Materials and methods), and some other genes, like *spoIIA*, reported to be induced during sporulation in *B*. *subtilis*. As shown in Fig 1, Spo0M homologs were not restricted to sporulating phyla like Firmicutes and Actinobacterias; they were also found in non-sporulating phyla, such as Euryarchaeota, Deinococcus, Chloroflexi, and Proteobacteria. It is interesting to note that in some species, such as *Exiguobacterium sp*. *AT1b*, *Exiguobacterium sibiricum 255–15*, *Macrococcus caseolyticus JCSC5402*, and *Thermincola potens JR*, the first three from the class *Bacilli* and the last one classified as *Clostridia*, in spite of having orthologs of Spo0A and Spo0M, the organism do not sporulate. Additionally, it is worth noting, that the rest of the orthologs of the sigma factors needed for the subsequent steps of sporulation are not present in these bacteria, so the presence of Spo0A may be related to other processes separate from sporulation. This observation, also suggests that the functional role of Spo0M should not only be restricted to sporulation (Fig 1) and opens the possibility of characterizing the function of Spo0M in depth.

### Spo0M null mutant displays morphological differences in cells grown in rich medium compared to wild type cells

**Fig 2:**
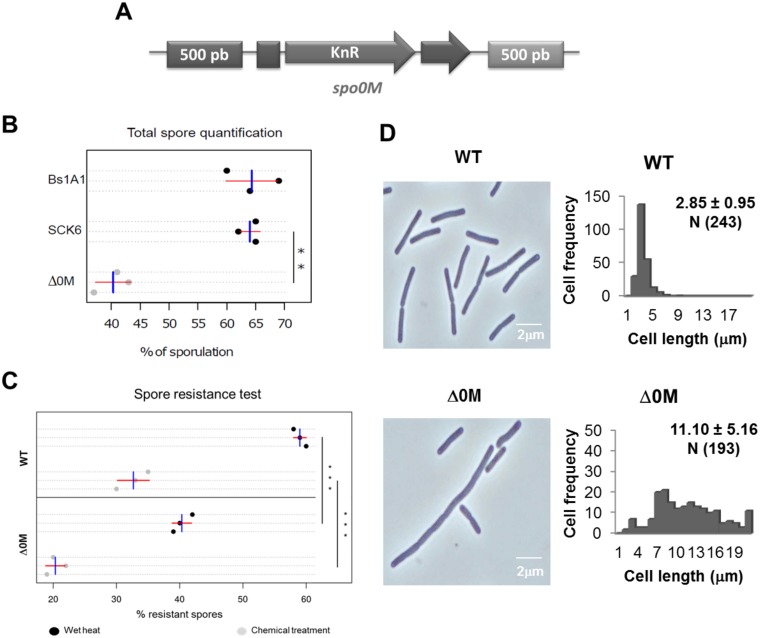
*spo0M* null mutant shows morphological alterations compared to the wild type strain. We generated a *spo0M* null mutant by allelic replacement of a pΔ0M construct (**A**) (see S1 File) in the *spo0M* gene. KnR, kanamycin resistance cassette. **B**. Spore quantification for the wild type and mutant strains. The wild type and mutant strains were cultured in Spizizen sporulation medium for 40 h. The total number of spores in each sample was quantified manually using a bright field microscope. The Δ0M strain generated 20–25% fewer spores than the wild type strain. The sporulation efficiency of the SCK6 parental strain was similar to that of the wild type Bs1A1 strain. **C**. Analysis of spore resistance in wild type and mutant strains. Spores of wild type and mutant strains were generated in 2XSG medium and subjected to wet heat and chemical treatment as mentioned in Materials and methods. Mutant spores are less resistant to both treatments, compared to those generated by the wild type strain. Data was analyzed through an ANOVA test, with a *p* value of 0.001, blue bar corresponds to the mean value and red bar represents the standard deviation. **D**. We analyzed the phenotype of the mutant strain using light microscopy and observed that the mutant adopted an elongated appearance compared with the wild type strain. Cell length was measured using the Fiji—ImageJ software [31].

A previously reported *spo0M* null mutant is blocked at stage zero of sporulation and is susceptible to lysis during vegetative growth [3]. However, it has not been reported whether this null mutant is defective in any other processes during vegetative growth or even if it is affected in other stages of sporulation. Therefore, we generated a *spo0M* null mutant (Δ0M) by introducing an antibiotic resistance cassette into the middle of the gene (Fig 2A) and then analyzed the physiology and phenotype of the resulting strain. We found that the growth rate of this mutant was not affected in either rich or sporulation media (S1A and S1B Fig); however, there was an effect with regard to the total number of spores generated by this strain (Fig 2B). Han *et al*. previously reported that a *spo0M* null mutant generated in the *B*. *subtilis* 168 strain has impaired sporulation ability, mainly in nutritionally rich medium (the mutant strain generates 90–99% fewer spores than the parental strain) but also in sporulation medium (generating 25–90% fewer spores than the parental strain in this case) [3]. Our results using the SCK6 strain are concordant with the referenced study: we observed a 20–25% decrease in sporulation in the mutant strain compared to the parental strain when grown in sporulation medium (Fig 2B). It should be noted that the total number of spores generated by the SCK6 strain was similar to the total number of spores generated by the wild type *B*. *subtilis* 168 strain (Bs1A1), which was evaluated in a previous report [3] (Fig 2B). Additionally, we assessed spore resistance to treatments with wet heat and chemicals (see Materials and methods). The spores generated by the *B*. *subtilis* Δ0M strain were less resistant to both treatments (Fig 2C). Heat resistance and resistance to chemical hazards are conferred upon the spore by the mineralization of its core, through the association of DNA to the Small Acid Soluble Proteins (SASPs) and by coat specific elements [49] that are expressed from σ^E^ and σ^K^ promoters from stage II of sporulation [49,50]. The fact that spores generated by the *spo0M* null mutant are less resistant than the wild type spores suggest that Spo0M could be involved in later stages of the sporulation process and not just at stage zero. Further analyses to determine how Spo0M might affect spore resistance are necessary.

Phenotypic analysis of Δ0M mutant cells showed that approximately 30% of these cells appeared relatively elongated compared to the parental strain when cultured in rich medium until exponential phase (OD_600_ 0.4–0.6; 1–3h of culture). Using the Fiji-ImageJ software [31], we determined that the cell length distribution of the Δ0M cells was significantly different (approximately two- to five-fold longer, being the mean value 11.10 ± 5.16 μm (N = 193)) to that of the wild type cells, (mean value of 2.85 ± 0.95 μm (N = 243)) (Fig 2D). The presence of elongated cells suggests impairment or a delay in cell division that leads to excessive cell growth before a normal mid-division generates two daughter cells.

**Fig 3:**
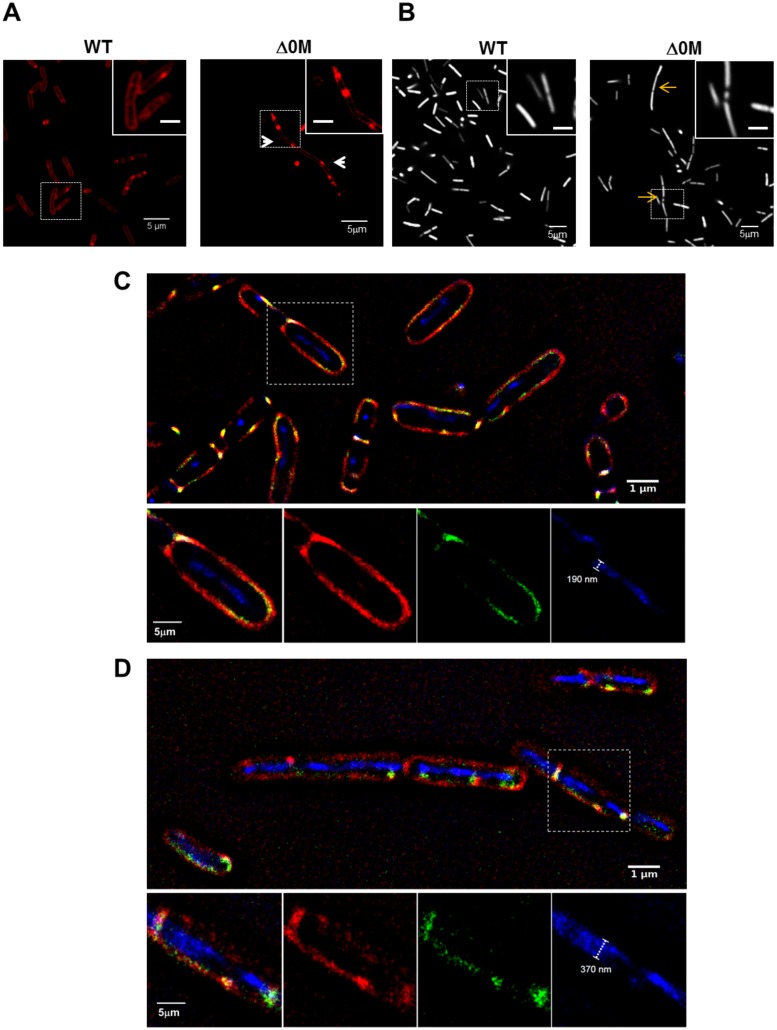
Δ0M mutant strain shows morphological alteration when compared with the wild type strain. **A**. The wild type and mutant cells were stained using the membrane dye FM4-64, and the cells were visualized by confocal microscopy. The *spo0M* null mutant cells had unique morphological features in their membranes, such as slight curvature (white arrow heads), which were not present in the wild type cells. **B**. The wild type and mutant cells were stained with the DNA marker Hoechst and observed by confocal microscopy. There are alterations in the DNA of the Δ0M strain, that appears to be decondensed or guillotined (orange arrows). Scale bar inside the box is 1μm. Cells from the GFP:ZapA and GFP:ZapA:Δ0M induced strains were stained with FM4-64 and Hoechst and were analyzed using super resolution microscopy **C**. Wild type cells. **D**. Δ0M mutant cells show elongated bacilli with containing several DNA packages and these appear decondensed, when measured and compared to the wild type cell DNA. Images were analyzed using the Fiji—ImageJ software [31].

We then stained the wild type and mutant cells with the membrane dye FM4-64 and the DNA marker Hoechst to further examine their structural elements. The mutant cells exhibited unique membrane morphology, showing apparent slight curvature and cell bending, which were not observed in the wild type cells (Fig 3A). Additionally, DNA structure in the cell is also altered, appearing as de-condensed or guillotined (Fig 3B).

Cell division is a complex process that begins with the formation of a septum and ends with the constriction of the Z ring. A large number of proteins and membrane elements are needed to accomplish the process [12,18]. As the establishment of the septum is a key step in cell division, we sought to determine whether Spo0M is involved in this process. To accomplish this, we analyzed septum formation and localization in wild type and Δ0M mutant cells using the fluorescent fusion protein GFP:ZapA in the FG347 strain (S2 Table). ZapA (Z ring-associated protein) is a membrane protein component of the divisome that is recruited during the early stages of septum formation; it stimulates FtsZ polymerization and stabilizes the divisome’s association with the membrane. Because ZapA colocalizes with FtsZ, it can be used as a septum marker [9].

Septum formation was analyzed by quantifying fluorescence intensity in an Amnis Image-Stream Mark II flow cytometer and data were processed using the IDEAS 6.1 software (Amnis Corporation). Septum formation rate was not altered in the Δ0M mutant cells and occurred in the same proportion as found in wild type cells (53.8 ± 4.4% of wild type cells formed a septum, whereas 51.8 ± 5.7% of mutant cells formed a septum under the tested conditions). We evaluated septum formation and localization using super-resolution microscopy (Fig 3C and 3D). Compared with wild type cells (Fig 3C) *spo0M* null mutation generates chains of bacilli of variable length, some of them containing several DNA packages, or one long DNA fiber, and these fibers have a de-condensed appearance (Fig 3D and S1 Video). Using the Fiji—ImageJ software [31], we measured the diameter of DNA fibers in wild type and mutant cells and found that the diameter of the DNA fibers present in the mutant cells is significantly greater than that found those of wild type cells (S2 Fig), which supports the hypothesis that DNA might be decondensed in the *spo0M* null mutants. While cell morphology is altered, however, septa are present even in the elongated cells, and therefore we assume that the *spo0M* null mutation affects the cell after the first stages of septum establishment. This result suggests that the Δ0M mutant cells, despite exhibiting correct septum formation, might have a reduced efficiency for septum positioning or constriction, a phenotype previously reported in *ftsA* mutants [8,51].

In the referenced *ftsA* mutants, the Z ring was well positioned, but its interaction with the cell membrane was not effective and constriction was impeded, and cell division was blocked [8,10]. We therefore hypothesize that Spo0M is involved in the cell division process during the vegetative growth of *Bacillus subtilis*, which opens new possibilities for the functional study of this protein.

### Intracellular localization of SpoM is dependent on the bacterial life cycle

**Fig 4:**
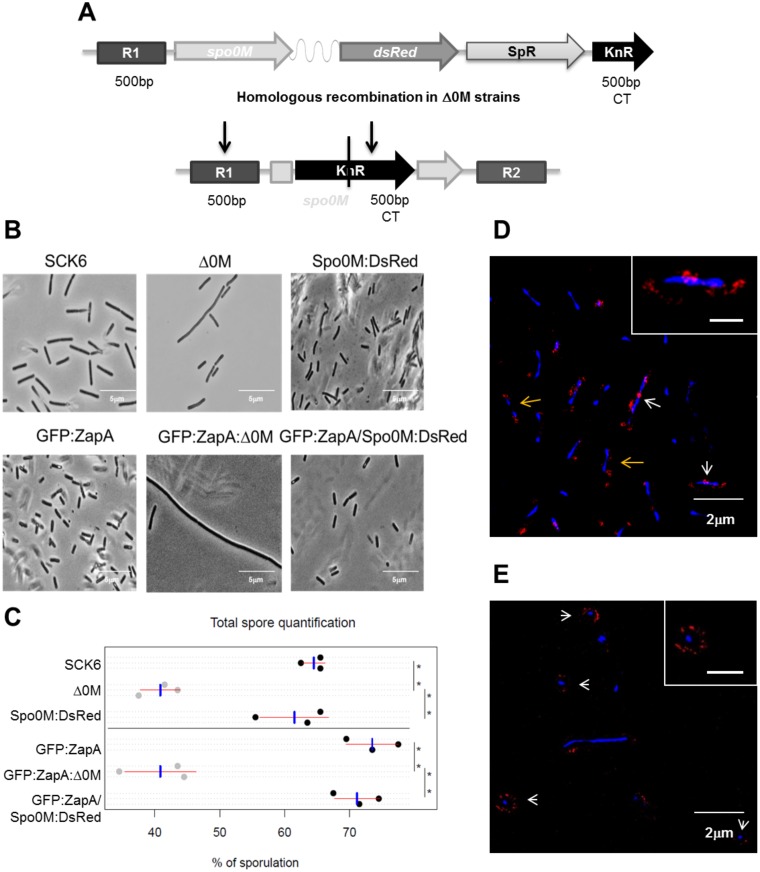
Spo0M exhibits a scattered distribution pattern in cells, appearing near the cell poles and the septum region. We generated a Spo0M:DsRed fusion protein via allelic replacement using the pSGGS construct **(A)** (see S1 File). R1, 500 bp upstream of the 5’ intergenic region of *spo0M*; SpR, spectinomycin resistance cassette; KnR, 500 bp of the CT region of a kanamycin resistance cassette. **B**. We analyzed the phenotypes of these strains using light microscopy and observed that the wild type phenotype was restored. The absence of elongated cells in the Spo0M:DsRed strains indicated that Spo0M functionality was complemented by the fusion protein **C**. Spore quantification of the wild type, mutant and Spo0M:DsRed strains. All of the strains were cultured in Spizizen sporulation medium for 40 h. The total number of spores in each sample was quantified manually using a bright field microscope. The number of spores generated by the Spo0M:DsRed strain was restored to wild type levels, validating that the Spo0M phenotype was complemented with the fusion protein. Data was analyzed through an ANOVA test and a Turkey multiple analysis of means, with a *p* value of 0.001, blue bar corresponds to the mean value and red bar represents the standard deviation. We used the fluorescent fusion protein to analyze the localization pattern of Spo0M during bacterial growth in rich and sporulation media. **D**. In rich medium (exponential phase of growth, 6–8 h of culture), Spo0M formed a scattered distribution near the cell poles (orange arrows) and in the central region of the cell, where a septum would be expected to form (white arrow). This distribution pattern remained until the appearance of the first spores (20–24 h). **E**. In sporulation medium (16–20 h of culture), Spo0M was localized to the forespores (arrow head), and it remained contained in these structures even after their release as mature spores (after 18–20 h). Scale bar inside the box is 1μm.

The expression and localization patterns of diverse proteins within a cell determine cell fate during differentiation. For example, after the onset of sporulation, the activation of different sigma factors in the mother cell and in the forespore regulates subsequent processes in each cell compartment by modulating the expression of specific proteins. To analyze the relationship between Spo0M function and its intracellular localization, we generated a Spo0M:DsRed fusion protein using the plasmid pSGGS (S2 Table) (Fig 4A). The translational fusion was integrated by homologous recombination in the Δ0M mutant strains (in the genetic background of the SCK6 and FG347strains), using 500 bp in the 5’ region of *spo0M* and 500 bp of the 3’ end of the kanamycine resistance cassette. We validated the chromosomal integration of the fusion gene construct using PCR (S3A and S3B Fig) and sequencing. The introduction of the Spo0M:DsRed construction did not cause any variation in the growth rate of the analyzed strains either in rich or sporulation media (S3C and S3D Fig). We also validated that the *spo0M*::*DsRed* gene fusion restored the wild type phenotype of the *B*. *subtilis* Δ0M strain by observation of bacterial morphology (Fig 4B) and by quantifying sporulation (Fig 4C). Cell morphology and cell length were restored in both strains containing the Spo0M:DsRed construction (Fig 4B). As mentioned previously, the average length of the wild type SCK6 cells was 2.85 ± 0.95 μm, whereas the average length of the Δ0M cells was 11.10 ± 5.16 μm. The cell length distribution of the Spo0M:DsRed cells was similar to that of the wild type cells, with a mean value of 2.58 ± 0.94 μm (N = 249), and it was significantly different than the cell length distribution of the mutant strain; we performed a multiple comparison test between each strain with a significance *p value* of 0.001 (S4 Fig). The sporulation efficiency of the Spo0M:DsRed strain was also nearly identical to that of the wild type strain (Fig 4C). Finally, we evaluated spore resistance to wet heat and chemical treatment in the strains containing the fusion protein. These results show that the spores generated by the Spo0M:DsRed strain had similar levels of resistance as those generated by the wild type strain (S3E Fig) and that the Spo0M:DsRed fusion protein complements the mutant phenotype. The reversion of the elongated phenotype supports the hypothesis that Spo0M is involved in the vegetative stage of growth of *B*. *subtilis* and possibly at the cell division process.

Using confocal and super-resolution microscopy, we analyzed Spo0M localization in bacteria grown in both rich and sporulation media over a 24 h period (S5A and S5B Fig). Bacterial culture in rich medium allowed us to assess Spo0M expression patterns during vegetative growth, while bacterial culture in sporulation medium facilitated the observation of cells during differentiation. We found that Spo0M:DsRed could be located throughout the cell periphery during vegetative growth, but with a tendency to accumulate near the cell poles and the central region of the cell (31% of the signal was present at the poles of the cells, and 37% of the signal was present in the central region; N = 456) (Fig 4D and S2 Video). The same distribution pattern was observed in the early stages of culture in both media and remained until the first spores appeared (24 h of culture in rich medium and 6–8 h of culture in sporulation medium). Upon the initiation of sporulation, Spo0M:DsRed accumulated in the forespore, and its concentration increased as spore maturation progressed (Fig 4E). Once the mature spore was released, after the lysis of the mother cell, a strong Spo0M:DsRed signal was still detectable.

The above results suggest that Spo0M localization depends on cell fate and might be related to Spo0M’s cellular functions. During vegetative growth, Spo0M is localized to the middle of the cell and at the cell poles, suggesting its involvement in a function other than sporulation control. This localization pattern supports our hypothesis that Spo0M might have a functional role during the vegetative stage growth, and in conjunction with all the observed results, suggests that Spo0M might be involved in cell division. During sporulation Spo0M accumulates in the spore until the spore matures and is released, suggesting that this protein acts not only at the beginning of the process. Several proteins involved in early stages of sporulation are later degraded by proteases when their function is no longer needed, as it happens with the sigma factors specific for each compartment [52,53] whereas, the proteins that remain in the spore are generally required for later stages or even for germination [54]. The presence of Spo0M in the spores suggests an additional function for this protein at later stages of sporulation.

### Spo0M is expressed in cells at early stages of vegetative growth

Analysis of Spo0M localization revealed detectable Spo0M:DsRed signals in cells at very early stages of growth, around the second hour of culture (S5A and S5B Fig). According to previous reports, *spo0M* transcription initiates from either a sigma factor σ^H^ or σ^W^ promoter [3,5,7]. The σ^H^ promoter is known to be expressed during the transition between the exponential phase of growth to the stationary phase [55], but it is also known that σ^H^ is an active vegetative sigma factor [56–58]; for example, it regulates the transcription of *ftsA* or *citGP2*, which are part of the SigmaH regulon and whose main transcription during the vegetative stage of growth is directed by this sigma factor [57,58].

Additionally, we determined that the promoter region of *spo0M* contains a predicted binding site for the housekeeping sigma factor σ^A^ (see Material and methods) (S6 Fig). The phenomenon of double regulation during the start of transcription has been previously observed in genes involved in sporulation, such as *spoVG*, which possesses two overlapping regulation sites, one a σ^H^ promoter and the other a regulatory binding site for AbrB [59], with both controlling expression during vegetative growth. It would be interesting to determine if *spo0M* is regulated via a similar mechanism.

To determine whether the observed fluorescent signal corresponded to the expression of Spo0M:DsRed, we further analyzed the expression of the fusion protein by Western blotting. The molecular weight of Spo0M is reported to be 28 kDa [3], which is close to the molecular weight of DsRed. Therefore, the molecular weight of the fusion protein is expected to be approximately 56 kDa. Our results showed that Spo0M:DsRed expression is detected from the first 2–4 h in culture in both rich and sporulation media. This expression stabilized around the tenth hour and essentially plateaued until the 22^nd^ hour (S5C and S5D Fig). This pattern is concordant with the results from microscopic analysis, in which the Spo0M:DsRed fusion protein was detected from early stages of growth in both media. These results reinforce the hypothesis that Spo0M is functional during the vegetative lifecycle of *B*. *subtilis*.

### Spo0M interacts with proteins involved in sporulation, signaling and cell division

Our next aim was to identify possible Spo0M interactors in *B*. *subtilis*. To accomplish this, we generated a FLAG-tagged Spo0M protein (Spo0M-FLAG) for use in immunoprecipitation assays performed using FLAG antibody-coupled agarose beads. We mixed cell extracts from the BL21-DE3 *E*. *coli* strain that had been induced with IPTG to express Spo0M-FLAG with cell extracts from the wild type *B*. *subtilis* 1A1 (Bs1A1) strain. As a control, we repeated the same experiment using the BL21-DE3 *E*. *coli* strain containing an empty FLAG vector. We loaded both samples onto an SDS-PAGE gel and then identified the proteins that appeared in the Spo0M-FLAG sample that differed from those in the control sample (S7 Fig). We obtained three biological replicates for this experiment. Selected bands were excised from the gel and digested, and the resultant peptides were analyzed by mass spectrometry.

**Fig 5:**
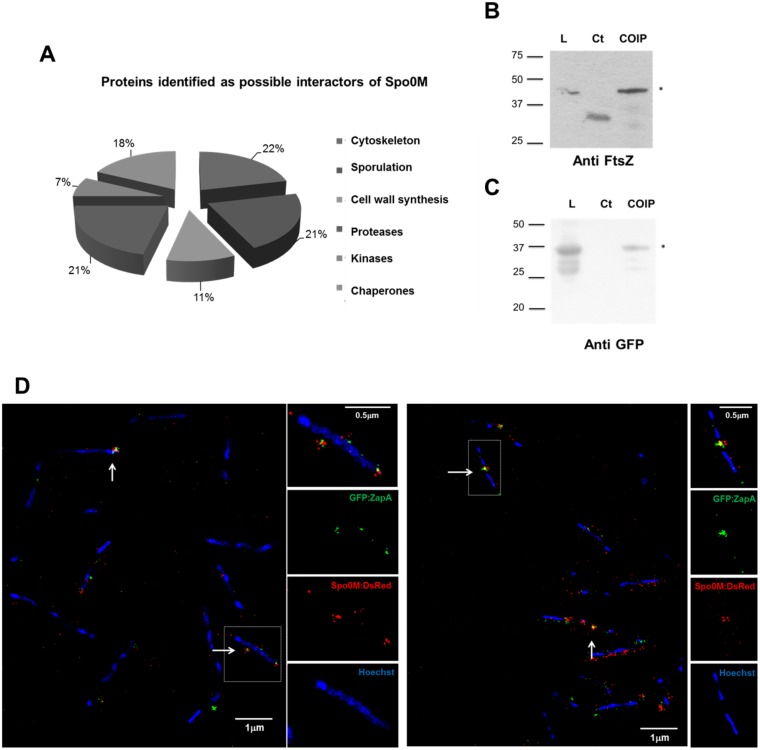
Spo0M interacting proteins are involved in sporulation, signaling and cell division. **A**. Pie chart showing the distribution of the identified proteins classified according to their functionality. We identified possible interaction partners of Spo0M by mass spectrometry analysis, using a FLAG-tagged empty vector as a control. **B**. Spo0M co-immunoprecipitates with FtsZ, indicating that it forms part of the same molecular complex. **C**. Spo0M also co-immunoprecipitates with GFP:ZapA, validating the interaction of Spo0M with some of the cell division proteins. Total protein extracts from a Spo0M:DsRed or an induced GFP:ZapZ/Spo0M:DsRed strains were allowed to interact with commercial anti-DsRed antibody and a protein A—sepharose column. FtsZ or GFP:ZapA precipitation was revealed by Western blotting using commercial anti-FtsZ or anti-GFP antibodies. As a control (Ct), we used the protein A—sepharose column with the total extract of protein mentioned above and an un-specific anti-IG antibody. L. Total extract of protein used in each experiment. **D**. Spo0M co-localizes with the cell division protein ZapA. Cells from the strain GFP:ZapA/Spo0M:DsRed were cultured in rich medium until the exponential phase (OD_600_ 0.4–0.6) and GFP:ZapA fluorescent fusion expression was induced with 0.1% xylose during 4h. DNA was stained with 0.01 mg mL^-1^ Hoechst (final concentration). The fusion proteins Spo0M:DsRed and GFP:ZapA co-localize in the medial region of the cell and near the cell poles (white arrows), corroborating that Spo0M interacts with a molecular complex of proteins involved in cell division.

As a result of this analysis, we identified proteases, kinases, chaperones, proteins involved in sporulation, cell division and cell wall synthesis (Fig 5A and S1 Table). Proteases, kinases and chaperones are common and often promiscuous components of protein interaction networks, however, it is remarkable that we found proteins that might be involved in sporulation, such as the ClpC/Clp-P protease system [60], PepF [61], Hrp kinase [62]. Meanwhile, the FtsH metalloprotease, involved in cell division [63], sporulation and biofilm formation [64,65], and as the only previously reported protein to interact with Spo0M [4] can be considered as an internal control for this experiment. In addition to the above mentioned proteins, in our immunoprecipitation experiment we also identified the chaperones GroEL, DnaK and trigger factor, which have been shown to generate filamentation when mutated in *E*. *coli* [66–68]. Further, it is known that DnaK directly interacts with FtsZ [67] and it has been reported that the lack of trigger factor in *B*. *subtilis* causes a delay in sporulation and germination [54].

Among the sporulation-related proteins that were enriched in our immunoprecipitation assay we encountered the master regulator Spo0A, whose expression levels fall when cells lack Spo0M [3]. However, it is not known whether the Spo0A-Spo0M interaction is direct, nor how Spo0M interferes with its expression. In addition to Spo0A, we also found proteins involved in later stages of sporulation, such as engulfment, coat and cortex assembly and germination (S1 Table). These results are in accordance with the results of the spore resistance tests reported in this work, which showed that the spores generated by the Δ0M strain are less resistant than those of the wild type strain and suggest an involvement of Spo0M at advanced stages of the differentiation process. Spo0M would not be the first example of a protein involved in more than one stage of sporulation; prior reports of similar behavior exist for the SpoIIE and SpoIIIE proteins. SpoIIE is a phosphatase essential for polar localization of the Z ring during asymmetrical division, where it associates with FtsZ [19]. After forespore formation, SpoIIE re-localizes to the internal side of the forespore membrane where it activates σ^F^, the first sigma factor specific to this structure, by releasing it from the SpoIIAB interaction [52]. On the other hand, SpoIIIE is a DNA translocase involved in DNA segregation during sporulation and some special cases of mid—division [69]; it also participates in the recombination process during dimer resolution in segregation, in a collaborative function with SftA [20,69]; and finally, it is involved in membrane fusion during engulfment [70]. Another example of a multi-functional protein is SpoVG. Mutants in *spovG* show impairment in asymmetrical division [71] and additionally, it is required for cortex synthesis [72]. If confirmed, Spo0M would join a family of proteins that exhibit multiple functions in different *B*. *subtilis* physiological contexts.

As part of this interactome, we found proteins involved in cell division and membrane dynamics, such as FtsZ [73], DivIVA [19], EzrA [11], FtsA [8], SepF [10] and FloA [74], and cell wall synthesis machinery, such as PBP1 [75], PBP3, PBP4 [76] and FtsE [14]. Mutants in all the aforementioned proteins involved in cell division generate a filamentous phenotype, for example, mutants in *ftsA*, the main protein that tethers the Z ring to the membrane, are extremely filamentous during vegetative growth and also show a reduction in sporulation [51]. A similar pattern is observed for mutants of *sepF*, which is believed to share functional redundancy with FtsA [10]. Mutants in *ezrA*, the negative regulator of FtsZ polymerization, show filamentation and formation of “mini cells” [11,77]; anucleated “mini cells” are also generated by a mutation in *divIVA* [78]; finally, certain mutations in the C-terminal end of *ftsZ* produce filaments, possibly due to the loss of interaction with other proteins involved in cell division [79]. FloA is a membrane organizer, an element of the bacterial lipid rafts, for which absence leads to alterations in cell morphology, competence, biofilm formation and sporulation [74,80,81]. In a similar way, mutants in cell wall synthesis machinery show filamentation and defects in membrane morphology similar to those reported for the *spo0M* null mutant in this current research [14,15,82]. Many of these factors have redundant functions in stabilizing the septum, promoting its constriction, or in regulating cell morphology and it is certain that there are still undiscovered elements that enable this machinery to function properly. The fact that the *spo0M* null mutation does not impair cell division or sporulation in its entirety, suggests that this protein has a redundant role with other bacterial protein(s) that precludes the development of more severe phenotypes.

The above results support the hypothesis for a role of Spo0M in the vegetative lifecycle of *B*. *subtilis*. According to this, we decided to corroborate the interaction of Spo0M with FtsZ, the main constituent of the Z ring and an essential element of this process, in both, mid—division and sporulation. For this assay, we mixed a total protein extract from the Spo0M:DsRed strain with an anti-DsRed antibody in a 1:100 ratio for 3h at 4°C. Next protein A coupled to sepharose beads was added to the mix and left to interact for 3h at 4°C. The beads were washed to eliminate non-interacting agents and were loaded onto an SDS-PAGE gel. We determined whether FtsZ coimmunoprecipitates with Spo0M:DsRed using Western blot analysis (Fig 5B and 5C). Our results showed that FtsZ is present in the same molecular complex as Spo0M:DsRed (Fig 5B), suggesting that Spo0M might be involved in cell division. We also performed the coimmunoprecipitation in the reverse order, wherein we mixed total protein extracts with a commercial anti-FtsZ antibody in a 1:100 ratio to purify FtsZ interactors using a protein A Sepharose column. As in the previous experiment, we separated the precipitated proteins by SDS-PAGE and determined whether Spo0M:DsRed coimmunoprecipitated with the FtsZ complex using an anti-DsRed antibody. The results corroborated the interaction between FtsZ and Spo0M (S8 Fig). To further validate the interaction of Spo0M with the molecular complex in which FtsZ is present during cell division, we evaluated the interaction of Spo0M with ZapA, another cell division protein that tethers FtsZ to the cell membrane and directly interacts with the Z ring [9]. We followed the same strategies described above for the coimmunoprecipitation assays with FtsZ, combining total protein extract from an induced GFP:ZapA/Spo0M:DsRed strain and an anti-GFP antibody. Although ZapA was not found as an enriched protein in the molecular complex coimmunoprecipitated with Spo0M, this might be explained due to its small size (9 kDa). In spite of this, we found that it does interact with Spo0M (Fig 5C), corroborating that Spo0M interacts with a molecular complex of proteins involved in cell division.

Finally, we analyzed the co-localization of the fusion proteins Spo0M:DsRed and GFP:ZapA using super-resolution microscopy. As shown in Fig 5D, Spo0M:DsRed co-localized with GFP:ZapA at the poles and mid-region of the cell (S3 and S4 Videos). The interaction of Spo0M with FtsZ and ZapA supports our hypothesis on the role of this protein as a protein involved in the cell division process of *B*. *subtilis* at the vegetative stage of life of this bacterium.

## Conclusions and future remarks

The present work provides unique insights supporting the hypothesis that Spo0M functions in cellular processes other than sporulation. Our results constitute the first evidence that Spo0M has a role in cell division during the vegetative growth stage. How does a *spo0M* mutation interfere with cell division? Do Spo0M-FtsZ and/or Spo0M-ZapA directly interact? Does this interaction occur during septum formation? Is this role in cell division also important during sporulation? Is Spo0M also participating in later stages of sporulation? There are still several open questions related to the functions of Spo0M in *B*. *subtilis*, however, the results presented here support the idea that Spo0M is not only a regulator of sporulation but also plays an important role during the vegetative growth of the bacterium. Our present efforts are currently focused on identifying proteins that interact with Spo0M, particularly those that are components of the bacterial cytoskeleton and membrane organizers. An improved understanding of the multifunctional role of Spo0M will allow a better understanding of the different cell processes in which Spo0M participate and how this processes are related.

## Supporting information

S1 File This file contains information of the plasmid construction methodology employed in this work and the list of the references mentioned in the Supporting Information. (DOCX)

S1 Fig Growth curves of the different strains used in this work. Growth curves for the different strains used in this work, listed in S2 Table, were determined in rich (**A**) and sporulation media (**B**). There were no significant differences in the growth rates of any of the strains in either rich or minimal media. The error bars represent standard deviations.
(TIF)

S2 Fig DNA fibers appear decondensed in the *spo0M* null mutant compared to the wild type strain. Using the Fiji—ImageJ software [31], we measured the diameter of DNA fibers in wild type and mutant cells and we found that the diameter of the DNA fibers present in the mutant cells is significantly longer than the one found in wild type cells. Data was analyzed by a Student´s T test of 2 tails assuming equal variances, the significance *p value* was 4.57 E-38; error bars represent the standard deviation.
(TIF)

S3 Fig Generation of the Spo0M-DsRed fluorescent fusion protein and localization of the protein over 24 h. **A**. and **B**. PCR amplification of DNA fragments for verification of the generation of the Spo0M-DsRed fusion strain in the SCK6 and FG347 genetic backgrounds. Chromosomal DNA was used for PCR amplification of genes. The following strains were used: A. SCK6/FG347, B. Δ0M/GFP:ZapA:Δ0M, C.Spo0M:DsRed/ GFP:ZapA/Spo0M:DsRed, and D. BsA1A. The expected gene sizes were *spo0M*, 950 bp; *Δ0M*, 1800 bp; *amyE*, 2100 bp; *amyE*:*gfp*:*zapA*, 4000 bp; and *dsRed*, 700 bp. Growth curves of the Spo0M:DsRed strains in rich (**C**) and minimal media (**D**). There were no significant differences in the growth rates of the analyzed strains in either rich or minimal media. The error bars represent standard deviations. **E**. Analysis of spore resistance in wild type, mutant and Spo0M:DsRed strains. The analysis was conducted as described in Materials and methods. The results show that the spores generated by the strain containing the fluorescent fusion protein have similar resistance to those generated by the wild type strain, which demonstrate that Spo0M function is reestablished within the Spo0M:DsRed strain. Data was analyzed through an ANOVA test and a Turkey multiple analysis of means, with a *p value* of 0.001, blue bar corresponds to the mean value and red bar represents the standard deviation.
(TIF)

S4 Fig Cell length distribution comparison between the wild type, Δ0M and Spo0M:DsRed strains. Cell length was measured using the Fiji—ImageJ software [31]. The cell length distribution of the Spo0M:DsRed cells was similar to that of the wild type cells and it was significantly different than the cell length distribution of the mutant strain; we performed a multiple comparison test between each strain with a significance *p value* of 0.001.
(TIF)

S5 Fig Spo0M is expressed from early stages of growth. We obtained samples of cells cultured and rich (**A**) or sporulation (**B**) media during 24h. Samples were taken each 2h; the cells were harvested and fixed with 4% PFA, then resuspended in 1X PBS and stained with the DNA marker DAPI at a final concentration of 0.01 mg mL^-1^. The cells were observed by confocal microscopy. **A**. In rich medium, Spo0M:DsRed is observed in a scattered pattern near the cell poles and the middle of the cell; the signal remains until the first spores appear, then the signal is concentrated in the spores. **B**. In sporulation medium, Spo0M:DsRed is directed to the forespores and it remains as an intense signal even after the spore is released. We obtained total protein extracts from bacterial samples cultured in rich and sporulation media. Samples were taken every 2 h for 22 h, and the expression of Spo0M was analyzed by Western blotting using a commercial DsRed antibody. **C**. Spo0M expression was detected at early stages of growth in rich medium: a DsRed signal was detected from the second hour of culture and reached a maximum concentration around the 10^th^ hour, remaining relatively constant until the 22^nd^ hour. **D**. Similar results were obtained in sporulation medium, in which Spo0M expression was detected from the 4^th^ hour of culture and then increased and remained constant until the 22^th^ hour.
(TIF)

S6 Fig *spo0M* orthologous genes have a consensus binding region for the vegetative stage sigma factor, σ^A^. 5´ intergenic sequences from *spo0M* orthologous genes were obtained and used to search for potential binding sites of the housekeeping σ^A^. The consensus sequence obtained was represented as a logo and corresponds with the σ^A^ promoter binding site sequence reported, 5′-TTGACA-17 nt-TATAAT-3′ [83].
(TIF)

S7 Fig Identification of the interaction elements of Spo0M in *B*. *subtilis*. A Spo0M-FLAG fusion protein was generated for the immunoprecipitation assays (see Material and methods). Cell extracts from the BL21-DE3 *E*. *coli* strain induced with IPTG to express Spo0M-FLAG were mixed with cell extracts of 8h, 10h and 12h culture of the Bs1A1 strain. The mixtures were made to interact with a column containing agarose beads coupled to an anti-FLAG antibody. As a control, we used the BL21-DE3 *E*. *coli* strain containing an empty FLAG vector. Problem and control samples were load onto an SDS-PAGE gel that was stained with Commassie Brilliant Blue and the differential bands in the problem samples with respect to the control were excised from the gel and digested; the resultant peptides were analyzed by mass spectrometry. The results represent the data of three biological replicates.
(TIF)

S8 Fig Spo0M coimmunoprecipitates with FtsZ. Total protein extracts from a Spo0M:DsRed strain were allowed to interact with commercial anti-FtsZ antibody in a protein A—sepharose column. Spo0M:DsRed precipitation was revealed by Western blot using commercial anti-DsRed antibody. As a control (Ct), we used the protein A—sepharose column with the total extract of Spo0M:DsRed protein and an non-specific anti-IG antibody. L. Total lysate of proteins of the Spo0M:DsRed strain.
(TIF)

S1 Table Possible interactors of Spo0M. We identified possible interaction partners of Spo0M by mass spectrometry analysis, using a FLAG-tagged empty vector as a control. The identified proteins are cited in this table, categorized by function.
(DOCX)

S2 Table Strains and plasmids used in this work. (DOCX)

S3 Table Oligonucleotides used in this work. (DOCX)

S1 Video Z stack animation of a *spo0M* null mutant cell. Cells from the GFP:ZapA:Δ0M strain were cultured in rich medium until the exponential phase (OD_600_ 0.4–0.6) and fluorescent fusion expression was induced with 0.1% xylose during 4h. Cells were harvested and resuspended in PBS 1X; membranes were stained with 0.01 mg mL^-1^ FM4-64 (final concentration) and DNA was stained with 0.01 mg mL^-1^ Hoechst (final concentration). Samples were analyzed using super resolution microscopy (see Materials and methods). Ten Z stack images were obtained and recorded as an animation video to show the entire cell. Mutant cells appear as chains of bacilli of variable length, with some alterations in membrane morphology and DNA of decondensed appearance.
(WMV)

S2 Video Z stack animation of a cell expressing Spo0M:DsRed. Cells from the Spo0M:DsRed strain were cultured in rich medium until the transition phase (OD_600_ 0.8–1.0). Cells were harvested and resuspended in PBS 1X and DNA was stained with 0.01 mg mL^-1^ Hoechst (final concentration). Samples were analyzed using super resolution microscopy (see Materials and methods). Ten Z stack images were obtained and recorded as an animation video to show the entire distribution of the Spo0M:DsRed fusion protein within the cell. Spo0M:DsRed appears in a scattered pattern in all the cell periphery, but more prevalent at the poles and medial region of the cell.
(AVI)

S3 Video Z stack animations of cells expressing the fluorescent fusions GFP:ZapA and Spo0M:DsRed. Cells from the GFP:ZapA/Spo0M:DsRed strain were cultured in rich medium until the exponential phase (OD_600_ 0.4–0.6) and GFP:ZapA fluorescent fusion expression was induced with 0.1% xylose during 4h. Cells were harvested and resuspended in PBS 1X and DNA was stained with 0.01 mg mL^-1^ Hoechst (final concentration). Samples were analyzed using super resolution microscopy (See Materials and Methods). Ten Z stack images were obtained and recorded as an animation video to show the entire distribution of the fusion proteins within the cells. GFP:ZapA and Spo0M:DsRed proteins co-localize in the mid region of the cell.
(AVI)

S4 Video Z stack animations of cells expressing the fluorescent fusions GFP:ZapA and Spo0M:DsRed. Cells from the GFP:ZapA/Spo0M:DsRed strain were cultured in rich medium until the exponential phase (OD_600_ 0.4–0.6) and GFP:ZapA fluorescent fusion expression was induced with 0.1% xylose during 4h. Cells were harvested and resuspended in PBS 1X and DNA was stained with 0.01 mg mL^-1^ Hoechst (final concentration). Samples were analyzed using super resolution microscopy (See Materials and Methods). Ten Z stack images were obtained and recorded as an animation video to show the entire distribution of the fusion proteins within the cells. GFP:ZapA and Spo0M:DsRed proteins co-localize in the cell poles.
(AVI)
