# Supplementary material for: Analysis of Spo0M function in Bacillus subtilis
Source: PLoS One. 2017 Feb 24;12(2):e0172737. doi: 10.1371/journal.pone.0172737 (PMC5325327; doi:10.1371/journal.pone.0172737)
Supplement: S1 File — (DOCX) [file pone.0172737.s001.docx]

**Supporting information**

**Plasmid construction**

All constructed plasmids were transformed into the DH5-α *E. coli* strain, after which they were transformed into the desired *B. subtilis* strain. The pUCm plasmid was constructed by deleting the *lacZ* and MCS regions from a pUC19 plasmid (D3404, Sigma-Aldrich) and replacing them with pUCmFw and pUCmRv oligonucleotides. These oligonucleotides contain restriction sites for PstI and BamHI restriction enzymes. To generate the pSpo0MFla plasmid, 500-bp regions flanking the *spo0M* gene were amplified by PCR using the above-described SpoFlaFw and SpoFlaRv oligonucleotides. The PCR product was cloned into the pUCm plasmid using the PstI and BamHI restriction sites. To construct the pΔ0M plasmid, the *spo0m* gene in the pSpoFla plasmid was disrupted by insertion of a kanamycin resistance cassette using internal restriction sites for HindIII and SacI. The pSGGS plasmid was synthetized by GenScript (Piscataway Township, NJ, USA). The pT7-Spo0M-FLAG plasmid was generated by inserting a *spo0M* PCR product with no stop codon sequence, which was obtained using the SpoXIF and SpoKIIR oligonucleotides (containing restriction sites for XhoI and KpnI, respectively), into a pT7-MAT-FLAG vector (Sigma).

**Supporting information references**

1. Turgay K, Persuh M, Hahn J, Dubnau D. Roles of the two ClpC atp binding sites in the regulation of competence and the stress response. Mol Microbiol. 2001;42: 717–727. doi:10.1046/j.1365-2958.2001.02623.x

2. Msadek T, Dartois V, Kunst F, Herbaud ML, Denizot F, Rapoport G. ClpP of Bacillus subtilis is required for competence development, motility, degradative enzyme synthesis, growth at high temperature and sporulation. Mol Microbiol. 1998;27: 899–914. doi:10.1046/j.1365-2958.1998.00735.x

3. Kang MS, Kim SR, Kwack P, Lim BK, Ahn SW, Rho YM, et al. Molecular architecture of the ATP-dependent CodWX protease having an N-terminal serine active site. Embo J. 2003;22: 2893–2902. doi:10.1093/emboj/cdg289

4. Frees D, Savijoki K, Varmanen P, Ingmer H. Clp ATPases and ClpP proteolytic complexes regulate vital biological processes in low GC, Gram-positive bacteria. Mol Microbiol. 2007;63: 1285–1295. doi:10.1111/j.1365-2958.2007.05598.x

5. Le ATT, Schumann W. The Spo0E phosphatase of Bacillus subtilis is a substrate of the FtsH metalloprotease. Microbiology. 2009;155: 1122–32. doi:10.1099/mic.0.024182-0

6. Prepiak P, Defrancesco M, Spadavecchia S, Mirouze N, Albano M, Persuh M, et al. MecA dampens transitions to spore, biofilm exopolysaccharide and competence expression by two different mechanisms. Mol Microbiol. 2011;80: 1014–1030. doi:10.1111/j.1365-2958.2011.07627.x

7. Thi Nguyen HB, Schumann W. The sporulation control gene spo0M of Bacillus subtilis is a target of the FtsH metalloprotease. Res Microbiol. Elsevier Masson SAS; 2012;163: 114–8. doi:10.1016/j.resmic.2011.10.011

8. Wehrl W, Niederweis M, Schumann W. The FtsH protein accumulates at the septum of Bacillus subtilis during cell division and sporulation. J Bacteriol. 2000;182: 3870–3873. doi:Doi 10.1128/Jb.182.13.3870-3873.2000

9. Kanamaru K, Stephenson S, Perego M. Overexpression of the PepF oligopeptidase inhibits sporulation initiation in Bacillus subtilis. J Bacteriol. 2002;184: 43–50. doi:10.1128/JB.184.1.43

10. Nessler S. The bacterial HPr kinase/phosphorylase: a new type of Ser/Thr kinase as antimicrobial target. Biochim Biophys Acta. 2005;1754: 126–131. doi:S1570-9639(05)00304-3 [pii]\r10.1016/j.bbapap.2005.07.042

11. Benson AK, Haldenwang WG. Bacillus subtilis sigma B is regulated by a binding protein (RsbW) that blocks its association with core RNA polymerase. Proc Natl Acad Sci U S A. 1993;90: 2330–4. Available: http://www.pubmedcentral.nih.gov/articlerender.fcgi?artid=46080&tool=pmcentrez&rendertype=abstract

12. Mogk A, Homuth G, Scholz C, Kim L, Schmid FX, Schumann W. The GroE chaperonin machine is a major modulator of the CIRCE heat shock regulon of Bacillus subtilis. EMBO J. 1997;16: 4579–90. doi:10.1093/emboj/16.15.4579

13. Sugimoto S, Saruwatari K, Higashi C, Sonomoto K. The proper ratio of GrpE to DnaK is important for protein quality control by the DnaK-DnaJ-GrpE chaperone system and for cell division. Microbiology. 2008;154: 1876–85. doi:10.1099/mic.0.2008/017376-0

14. Castanié-Cornet MP, Bruel N, Genevaux P. Chaperone networking facilitates protein targeting to the bacterial cytoplasmic membrane. BBA - Mol Cell Res. The Authors; 2014;1843: 1442–1456. doi:10.1016/j.bbamcr.2013.11.007

15. Schulz A, Tzschaschel B, Schumann W. Isolation and analysis of mutants of the dnaK operon of Bacillus subtilis. Mol Microbiol. 1995;15: 421–429. doi:10.1111/j.1365-2958.1995.tb02256.x

16. Guthrie B, Wickner W. Trigger factor depletion or overproduction causes defective cell division but does not block protein export. J Bacteriol. 1990;172: 5555–5562.

17. Baram D, Pyetan E, Sittner A, Auerbach-Nevo T, Bashan A, Yonath A. Structure of trigger factor binding domain in biologically homologous complex with eubacterial ribosome reveals its chaperone action. Proc Natl Acad Sci U S A. 2005;102: 12017–12022. doi:10.1073/pnas.0505581102

18. Wahlstrom E. The extracytoplasmic folding factor PrsA is required for protein secretion only in the presence of the cell wall in Bacillus subtilis. Microbiology. 2003;149: 569–577. doi:10.1099/mic.0.25511-0

19. Hyyryläinen H-L, Marciniak BC, Dahncke K, Pietiäinen M, Courtin P, Vitikainen M, et al. Penicillin-binding protein folding is dependent on the PrsA peptidyl-prolyl cis-trans isomerase in Bacillus subtilis. Mol Microbiol. 2010;77: 108–27. doi:10.1111/j.1365-2958.2010.07188.x

20. Driks A, Eichenberger P. The Spore Coat. ASMscience. 2016;4. doi:10.1128/microbiolspec.TBS

21. Yudkin MD, Harrison D. Effect of precisely identified mutations in the spoIIAC gene of Bacillus subtilis on the toxicity of the sigma-like gene product to Escherichia coli. Mol Gen Genet. 1987;209: 333–4. Available: http://www.ncbi.nlm.nih.gov/pubmed/3118147

22. McKenney PT, Eichenberger P. Dynamics of spore coat morphogenesis in Bacillus subtilis. Mol Microbiol. 2012;83: 245–60. doi:10.1111/j.1365-2958.2011.07936.x

23. Ozin AJ, Henriques AO, Yi H, Jr CPM. Morphogenetic Proteins SpoVID and SafA Form a Complex during Assembly of the Bacillus subtilis Spore Coat Morphogenetic Proteins SpoVID and SafA Form a Complex during Assembly of the Bacillus subtilis Spore Coat. J Bacteriol. 2000;182: 1828–1833. doi:10.1128/JB.182.7.1828-1833.2000.Updated

24. Steil L, Serrano M, Henriques AO, Völker U. Genome-wide analysis of temporally regulated and compartment-specific gene expression in sporulating cells of Bacillus subtilis. Microbiology. 2005;151: 399–420. doi:10.1099/mic.0.27493-0

25. Chen Y, Ray WK, Helm RF, Melville SB, Popham DL. Levels of germination proteins in Bacillus subtilis dormant, superdormant, and germinating spores. PLoS One. 2014;9: :e95781. doi: 10.1371/journal.pone.0095781. eColle. doi:10.1371/journal.pone.0095781

26. Imamura D, Kuwana R, Takamatsu H, Watabe K. Localization of proteins to different layers and regions of Bacillus subtilis spore coats. J Bacteriol. 2010;192: 518–24. doi:10.1128/JB.01103-09

27. Piggot PJ, Hilbert DW. Sporulation of *Bacillus subtilis*. Curr Opin Microbiol. 2004;7: 579–586. doi:10.1016/j.mib.2004.10.001

28. Beall B, Moran CP. Cloning and characterization of spoVR, a gene from Bacillus subtilis involved in spore cortex formation. J Bacteriol. 1994;176: 2003–2012. Available: http://www.pubmedcentral.nih.gov/articlerender.fcgi?artid=205306&tool=pmcentrez&rendertype=abstract

29. Scheffers DJ, Jones LJF, Errington J. Several distinct localization patterns for penicillin-binding proteins in Bacillus subtilis. Mol Microbiol. 2004;51: 749–764. doi:10.1046/j.1365-2958.2003.03854.x

30. Foulquier E, Pompeo F, Bernadac A, Espinosa L, Galinier A. The YvcK protein is required for morphogenesis via localization of PBP1 under gluconeogenic growth conditions in Bacillus subtilis. Mol Microbiol. 2011;80: 309–18. doi:10.1111/j.1365-2958.2011.07587.x

31. Erickson HP. FtsZ, a prokaryotic homolog of tubulin? Cell. 1995;80: 367–370. doi:10.1016/0092-8674(95)90486-7

32. Eswaramoorthy P, Erb ML, Gregory JA, Silverman J, Pogliano K, Pogliano J, et al. Cellular architecture mediates DivIVA ultrastructure and regulates min activity in Bacillus subtilis. MBio. 2011;2. doi:10.1128/mBio.00257-11

33. Land AD, Luo Q, Levin PA. Functional domain analysis of the cell division inhibitor EzrA. PLoS One. 2014;9: e102616. doi:10.1371/journal.pone.0102616

34. Pichoff S, Lutkenhaus J. Tethering the Z ring to the membrane through a conserved membrane targeting sequence in FtsA. Mol Microbiol. 2005;55: 1722–1734. doi:10.1111/j.1365-2958.2005.04522.x

35. Beall B, Lutkenhaus J. Impaired cell division and sporulation of a Bacillus subtilis strain with the ftsA gene deleted. J Bacteriol. 1992;174: 2398–2403. Available: http://www.ncbi.nlm.nih.gov/entrez/query.fcgi?cmd=Retrieve&db=PubMed&dopt=Citation&list_uids=1551857%5Cnhttp://www.ncbi.nlm.nih.gov/pmc/articles/PMC205866/pdf/jbacter00073-0356.pdf

36. Jones LJF, Carballido-López R, Errington J. Control of cell shape in bacteria: Helical, actin-like filaments in Bacillus subtilis. Cell. 2001;104: 913–922. doi:10.1016/S0092-8674(01)00287-2

37. Esue O, Cordero M, Wirtz D, Tseng Y. The assembly of MreB, a prokaryotic homolog of actin. J Biol Chem. 2005;280: 2628–2635. doi:10.1074/jbc.M410298200

38. Duman R, Ishikawa S, Celik I, Strahl H, Ogasawara N, Troc P, et al. Structural and genetic analyses reveal the protein SepF as a new membrane anchor for the Z ring. Proc Natl Acad Sci. 2013;110: E4601–E4610. doi:10.1073/pnas.1313978110

39. Yepes A, Schneider J, Mielich B, Koch G, García-Betancur J-C, Ramamurthi KS, et al. The biofilm formation defect of a Bacillus subtilis flotillin-defective mutant involves the protease FtsH. Mol Microbiol. 2012;86: 457–71. doi:10.1111/j.1365-2958.2012.08205.x

40. Donovan C, Bramkamp M. Characterization and subcellular localization of a bacterial flotillin homologue. Microbiology. 2009;155: 1786–1799. doi:10.1099/mic.0.025312-0

41. Garti-Levi S, Hazan R, Kain J, Fujita M, Ben-Yehuda S. The FtsEX ABC transporter directs cellular differentiation in Bacillus subtilis. Mol Microbiol. 2008;69: 1018–28. doi:10.1111/j.1365-2958.2008.06340.x

42. Burkholder PR, Giles NH. Induced biochemical mutations in Bacillus subtilis. Am J Bot. 1947;34: 345–348. doi:10.2307/2437147

43. Zhang XZ, Zhang YHP. Simple, fast and high-efficiency transformation system for directed evolution of cellulase in Bacillus subtilis. Microb Biotechnol. 2011;4: 98–105. doi:10.1111/j.1751-7915.2010.00230.x

44. Gueiros-Filho FJ, Losick R. A widely conserved bacterial cell division protein that promotes assembly of the tubulin-like protein FtsZ. Genes Dev. 2002;16: 2544–2556. doi:10.1101/gad.1014102
