# Supplementary figures and images for: Analysis of Spo0M function in Bacillus subtilis
Source: PLoS One. 2017 Feb 24;12(2):e0172737. doi: 10.1371/journal.pone.0172737 (PMC5325327; doi:10.1371/journal.pone.0172737)

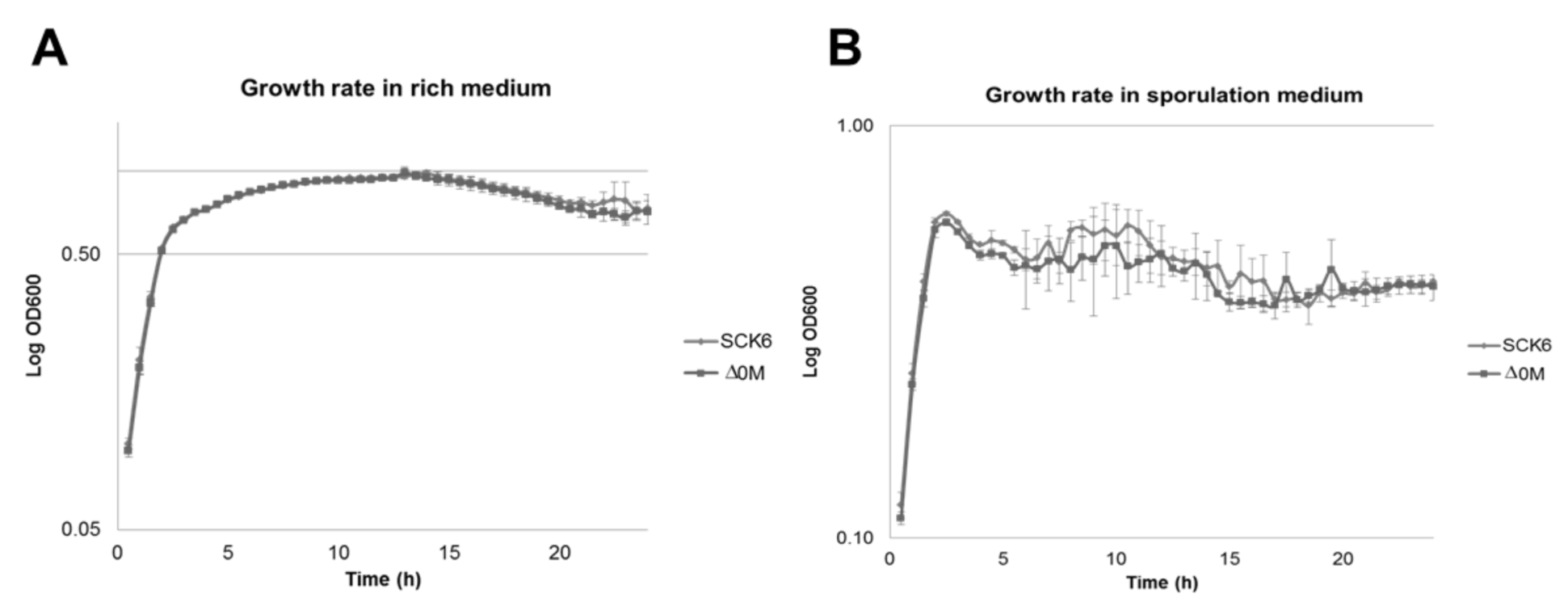

Supplement: S1 Fig — Growth curves for the different strains used in this work, listed in S2 Table, were determined in rich (A) and sporulation media (B). There were no significant differences in the growth rates of any of the strains in either rich or minimal media. The error bars represent standard deviations. (TIF) [file pone.0172737.s002.tif]

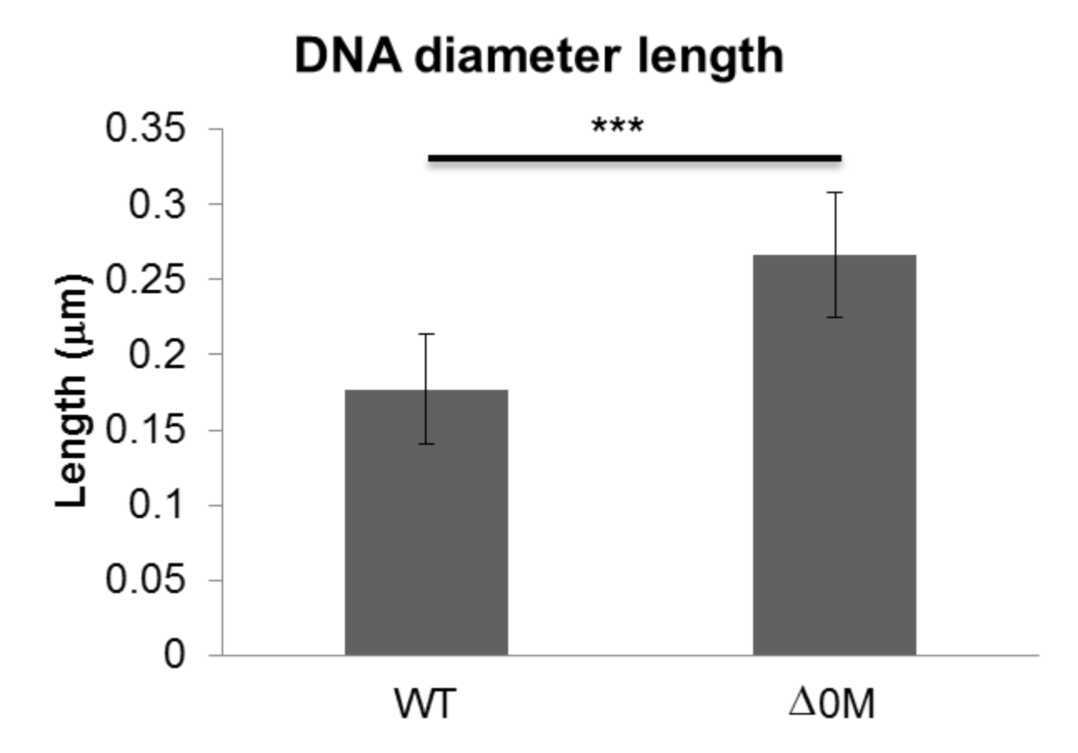

Supplement: S2 Fig — Using the Fiji—ImageJ software [31], we measured the diameter of DNA fibers in wild type and mutant cells and we found that the diameter of the DNA fibers present in the mutant cells is significantly longer than the one found in wild type cells. Data was analyzed by a Student´s T test of 2 tails assuming equal variances, the significance p value was 4.57 E-38; error bars represent the standard deviation. (TIF) [file pone.0172737.s003.tif]

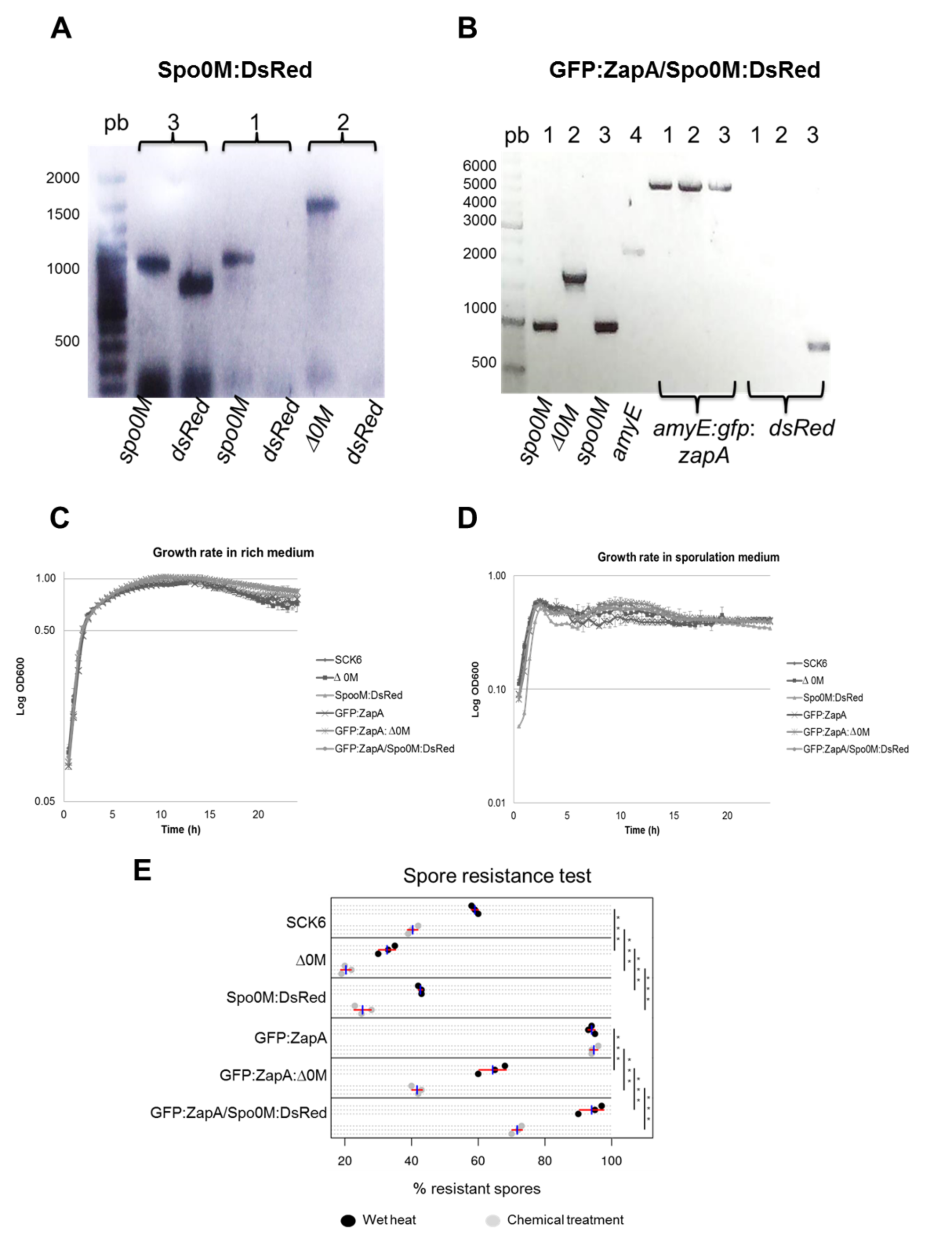

Supplement: S3 Fig — A. and B. PCR amplification of DNA fragments for verification of the generation of the Spo0M-DsRed fusion strain in the SCK6 and FG347 genetic backgrounds. Chromosomal DNA was used for PCR amplification of genes. The following strains were used: A. SCK6/FG347, B. Δ0M/GFP:ZapA:Δ0M, C.Spo0M:DsRed/ GFP:ZapA/Spo0M:DsRed, and D. BsA1A. The expected gene sizes were spo0M, 950 bp; Δ0M, 1800 bp; amyE, 2100 bp; amyE:gfp:zapA, 4000 bp; and dsRed, 700 bp. Growth curves of the Spo0M:DsRed strains in rich (C) and minimal media (D). There were no significant differences in the growth rates of the analyzed strains in either rich or minimal media. The error bars represent standard deviations. E. Analysis of spore resistance in wild type, mutant and Spo0M:DsRed strains. The analysis was conducted as described in Materials and methods. The results show that the spores generated by the strain containing the fluorescent fusion protein have similar resistance to those generated by the wild type strain, which demonstrate that Spo0M function is reestablished within the Spo0M:DsRed strain. Data was analyzed through an ANOVA test and a Turkey multiple analysis of means, with a p value of 0.001, blue bar corresponds to the mean value and red bar represents the standard deviation. (TIF) [file pone.0172737.s004.tif]

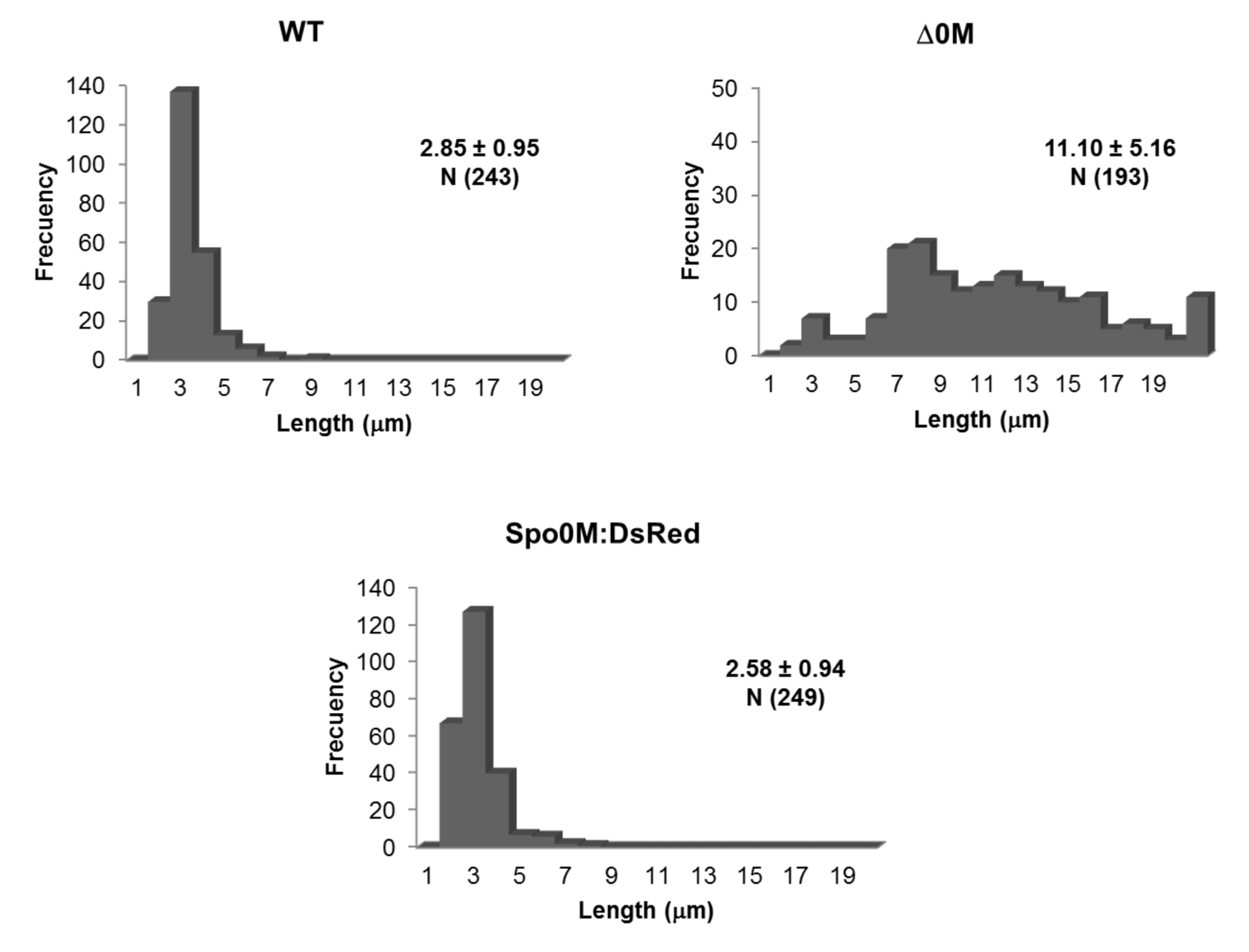

Supplement: S4 Fig — Cell length was measured using the Fiji—ImageJ software [31]. The cell length distribution of the Spo0M:DsRed cells was similar to that of the wild type cells and it was significantly different than the cell length distribution of the mutant strain; we performed a multiple comparison test between each strain with a significance p value of 0.001. (TIF) [file pone.0172737.s005.tif]

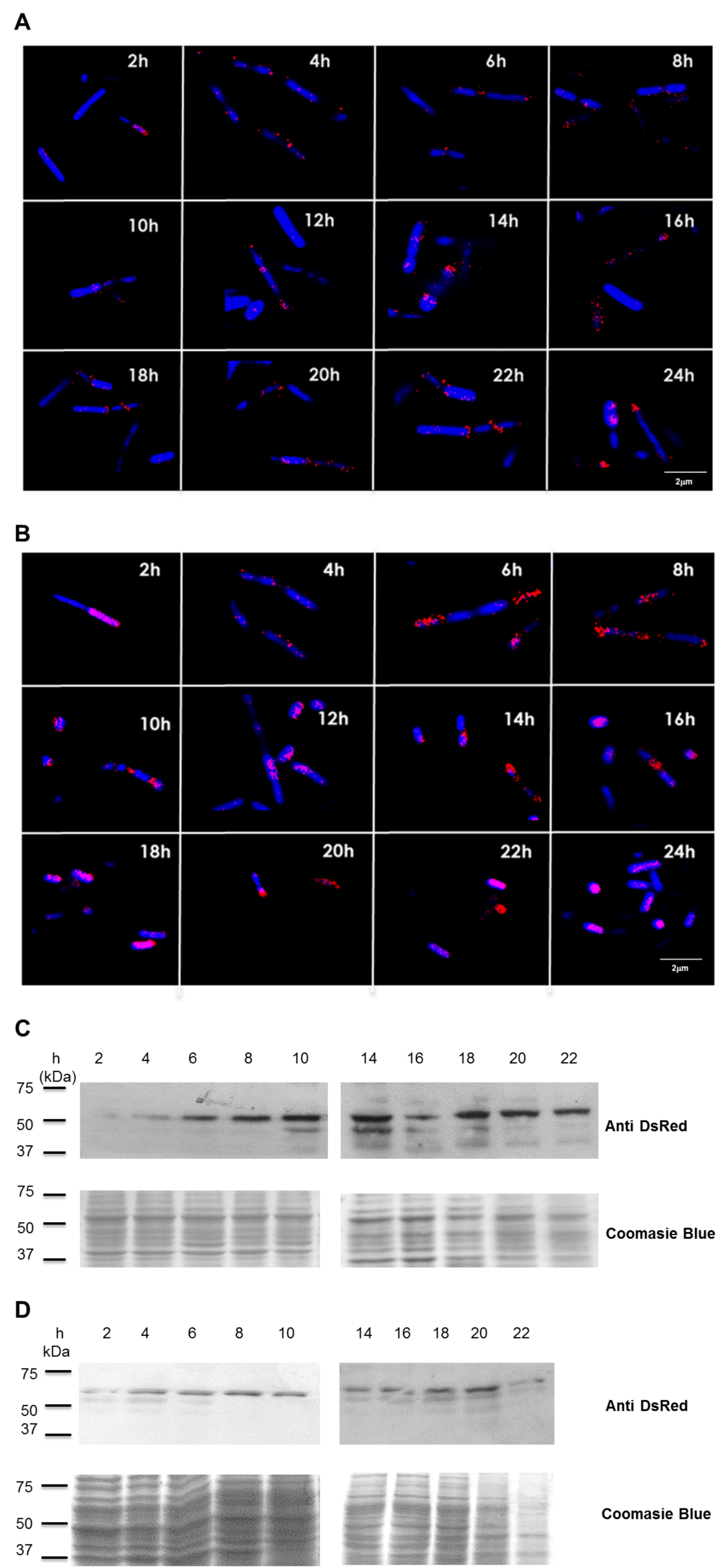

Supplement: S5 Fig — We obtained samples of cells cultured and rich (A) or sporulation (B) media during 24h. Samples were taken each 2h; the cells were harvested and fixed with 4% PFA, then resuspended in 1X PBS and stained with the DNA marker DAPI at a final concentration of 0.01 mg mL-1. The cells were observed by confocal microscopy. A. In rich medium, Spo0M:DsRed is observed in a scattered pattern near the cell poles and the middle of the cell; the signal remains until the first spores appear, then the signal is concentrated in the spores. B. In sporulation medium, Spo0M:DsRed is directed to the forespores and it remains as an intense signal even after the spore is released. We obtained total protein extracts from bacterial samples cultured in rich and sporulation media. Samples were taken every 2 h for 22 h, and the expression of Spo0M was analyzed by Western blotting using a commercial DsRed antibody. C. Spo0M expression was detected at early stages of growth in rich medium: a DsRed signal was detected from the second hour of culture and reached a maximum concentration around the 10th hour, remaining relatively constant until the 22nd hour. D. Similar results were obtained in sporulation medium, in which Spo0M expression was detected from the 4th hour of culture and then increased and remained constant until the 22th hour. (TIF) [file pone.0172737.s006.tif]

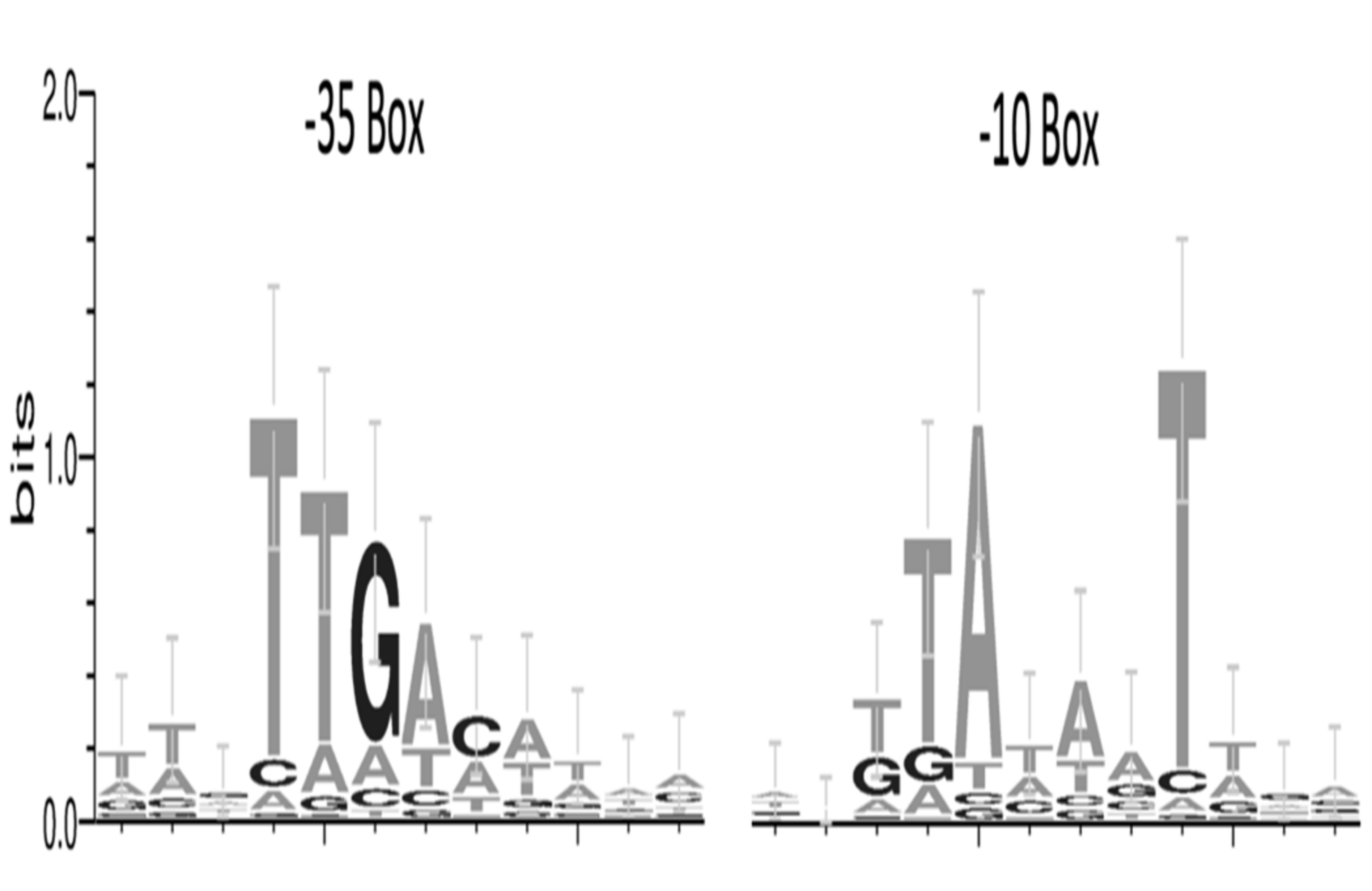

Supplement: S6 Fig — 5´ intergenic sequences from spo0M orthologous genes were obtained and used to search for potential binding sites of the housekeeping σA. The consensus sequence obtained was represented as a logo and corresponds with the σA promoter binding site sequence reported, 5′-TTGACA-17 nt-TATAAT-3′ [83]. (TIF) [file pone.0172737.s007.tif]

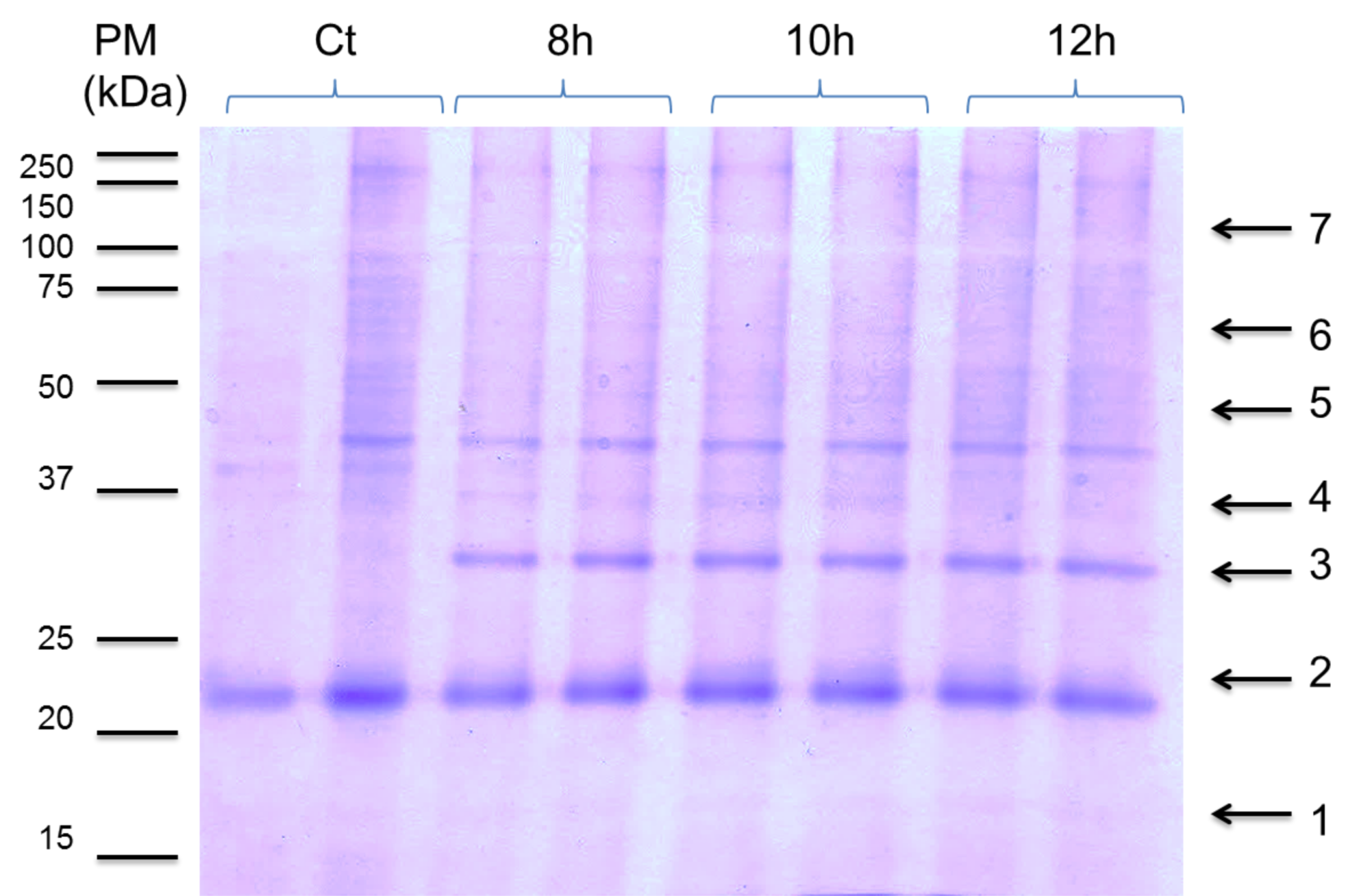

Supplement: S7 Fig — A Spo0M-FLAG fusion protein was generated for the immunoprecipitation assays (see Material and methods). Cell extracts from the BL21-DE3 E. coli strain induced with IPTG to express Spo0M-FLAG were mixed with cell extracts of 8h, 10h and 12h culture of the Bs1A1 strain. The mixtures were made to interact with a column containing agarose beads coupled to an anti-FLAG antibody. As a control, we used the BL21-DE3 E. coli strain containing an empty FLAG vector. Problem and control samples were load onto an SDS-PAGE gel that was stained with Commassie Brilliant Blue and the differential bands in the problem samples with respect to the control were excised from the gel and digested; the resultant peptides were analyzed by mass spectrometry. The results represent the data of three biological replicates. (TIF) [file pone.0172737.s008.tif]

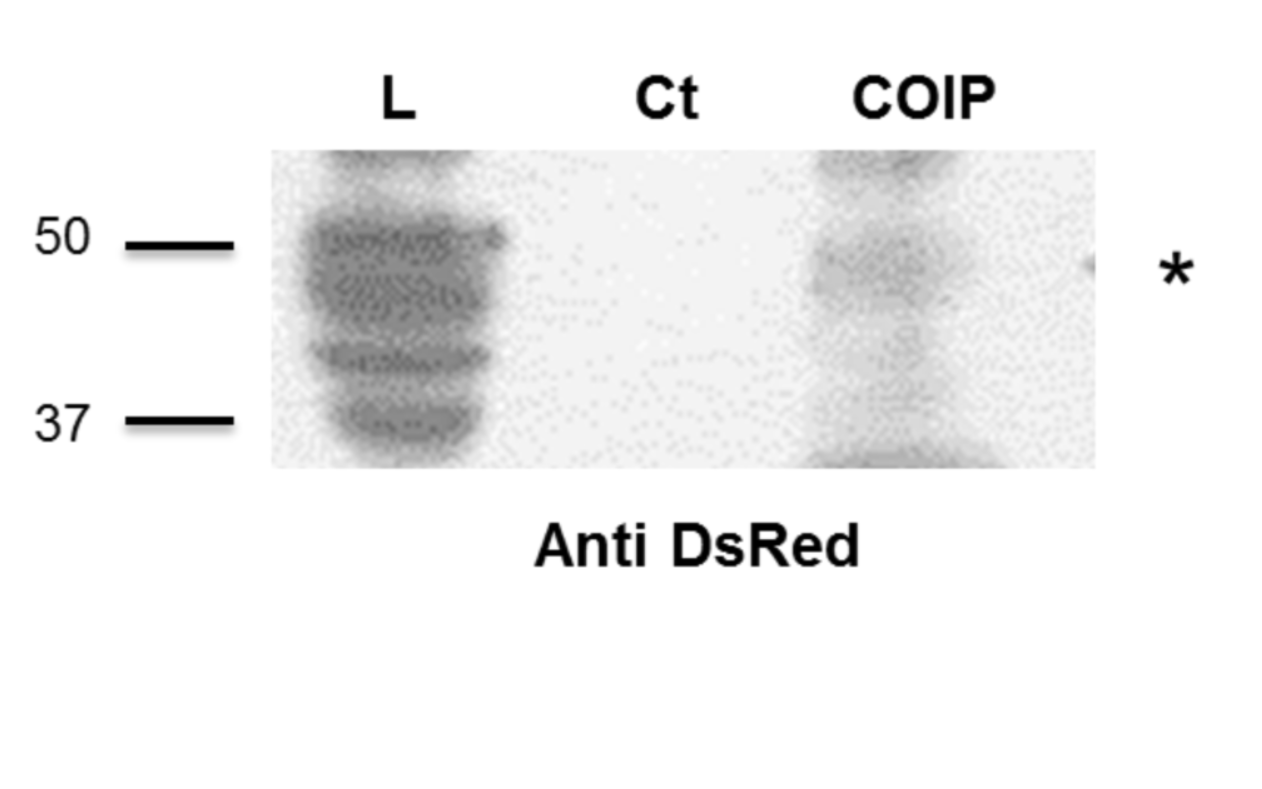

Supplement: S8 Fig — Total protein extracts from a Spo0M:DsRed strain were allowed to interact with commercial anti-FtsZ antibody in a protein A—sepharose column. Spo0M:DsRed precipitation was revealed by Western blot using commercial anti-DsRed antibody. As a control (Ct), we used the protein A—sepharose column with the total extract of Spo0M:DsRed protein and an non-specific anti-IG antibody. L. Total lysate of proteins of the Spo0M:DsRed strain. (TIF) [file pone.0172737.s009.tif]
