# Supplementary material for: Analysis of Spo0M function in Bacillus subtilis
Source: PLoS One. 2017 Feb 24;12(2):e0172737. doi: 10.1371/journal.pone.0172737 (PMC5325327; doi:10.1371/journal.pone.0172737)
Supplement: S1 Table — We identified possible interaction partners of Spo0M by mass spectrometry analysis, using a FLAG-tagged empty vector as a control. The identified proteins are cited in this table, categorized by function. (DOCX) [file pone.0172737.s010.docx]

S1 Table

| **Supplementary Table 1. Possible interactors of Spo0M** | |
| --- | --- |
| **Name** | **Function** |
| *Proteases* |  |
| ClpC | ATPase subunit of ATP-dependent protease; serine protease [1]. |
| ClpP | Proteolytic subunit of ATP-dependent proteases; protein quality control in processes like competence, sporulation and motility [2,3]. |
| ClpY | ATPase subunit of ATP-dependent protease; serine protease [4]. |
| ClpQ | ATP-dependent protease, belonging to a two component system ClpQ/ClpY [3]. |
| FtsH | ATP-dependent metalloprotease, involved in cell division, sporulation and biofilm formation [5,6]. Degrades Spo0E and Rap phosphatases [5]. Degrades Spo0M [7]. Accumulates in the septum during cell division and sporulation [8]. |
| PepF | Oligoendopeptidase involved in normal protein turnover. Overexpression causes delay in the onset of sporulation and competence [9]. |
| *Kinases* |  |
| HPrK | Serine kinase/phosphorylase involved in carbon catabolite repression [10]. |
| RsbW | Serine kinase, regulates sigma B-dependent transcription [11]. |
| *Repair machinery/Chaperones* | |
| GroEL | Chaperonin belonging to the HSP60 family, induced by heat shock; co-repressor for HrcA [12]. Lack of GroEL in *E. coli* generates filamentous cells [13]. |
| DnaK | Class I heat shock protein, function in the folding of nascent peptides [14]. Downregulation of DnaK in *E. coli* causes filamentation and delocalization of FtsZ [13,15]. |
| Trigger factor | Chaperone that interacts with the newly synthetized peptides; lack of *tig* generates filamentation [16]. Lack in *B. subtilis* causes a delay in spore germination [17]. |
| PrsA | Molecular chaperone that works post-translocation of exported proteins [18]. Required for folding of PBP's [19]. |
| *Sporulation* |  |
| Spore coat protein E | Required to the coat assembly [20]. |
| SpoIIAC | Present in the mother cell, necessary for the degradation of the cell wall after septum formation and before engulfment [21]. |
| SpoIIIAH | Part of the transmembrane region that joins the mother cell to the forespore, necessary to the SigmaG expression and the coat assembly [22]. |
| SpoIVD asociated factor A | Probably involved in coat protein assembly, related to lysozyme resistance; also involved in germination [23]. |
| YdcC | Unknown function. A null mutant shows defects in sporulation and in SigmaG expression. Probably involved in engulfment [24]. |
| GerM | Hydrolysis of the cortex during germination [25,26]. |
| GerKA | Sugar, aspartate and KCl receptor during germination [25,26]. |
| GerQ | Necessary for germination [25,26]. |
| Spo0A | Master regulator of sporulation, its phosphorylation determines the onset of the process [27]. |
| Spore coat protein F | Involved in spore resistance [20]. |
| SpoVR | Involved in cortex synthesis [28]. |
| *Cell wall synthesis machinery* | |
| PBP3 | Cell wall synthesis, probably involved in cell elongation [29]. |
| PBP1 | Cell wall synthesis during cell division [30]. |
| PBP4 | Cell wall synthesis, probably involved in cell elongation [29]. |
| *Cytoskeleton and membrane dynamics* | |
| FtsZ | Analog of the eukaryotic tubulin, main component of the septum. It is indispensable for cell division and sporulation [31]. |
| DivIVA | Avoid formation of aberrant septum in places where the chromosome is present; is located in areas of cell division in early stages and forms rings next to the septum that remains until the end of cytokinesis [32]. |
| EzrA | Negative regulator of FtsZ polymerization, avoid its location in unspecific areas and maintain an available pool of the protein [33]. |
| FtsA | Member of the MreB family of proteins, necessary for the stabilization of Z ring in the membrane and the recruitment of other elements of the divisome [34,35]. |
| MreB | Homolog of the eukaryotic actin, essential for the maintenance of the cell shape; directs the synthesis of the cell wall [36,37]. |
| SepF | Tethers FtsZ to the cell membrane during cell division, directs the movements of small filaments of FtsZ through the membrane [38]. |
| FloA | Flotillin homolog; involved in lipid raft and membrane organization. When mutated it affects sporulation [39], biofilm formation, competence and cell morphology [40]. |
| FtsE | ABC transporter part of the two component system FtsEX, necessary for cell wall elongation and phosphorylation of Spo0A [41]. |
