# Supplementary material for: Analysis of Spo0M function in Bacillus subtilis
Source: PLoS One. 2017 Feb 24;12(2):e0172737. doi: 10.1371/journal.pone.0172737 (PMC5325327; doi:10.1371/journal.pone.0172737)
Supplement: S2 Table — (DOCX) [file pone.0172737.s011.docx]

S2 Table

| Strain/Plasmid | Features | References |
| --- | --- | --- |
| *Strains* |  |  |
| BS1A1 | Wild type *B. subtilis* 168 derivative | [42] |
| SCK6 | Em his nprE18 aprE3 eglS(DELTA)102 bglT/bglS(DELTA)EV *lacA::PxylA-comK* | [43] |
| Δ0M | *lacA::PxylA-comK (erm), spo0MD::kan* | This work |
| Spo0M:DsRed | *lacA::PxylA-comK (erm), spo0M-dsred (sp)* | This work |
| FG347/GFP:ZapA | *amyE::Pxyl-gfp-zapA (cat)* | [44] |
| GFP:ZapA:Δ0M | *amyE::Pxyl-gfp-zapA (cat), spo0MD::kan* | This work |
| GFP:ZapA/Spo0M:DsRed | *amyE::Pxyl-gfp-zapA (cat), spo0M-dsred (sp)* | This work |
| *Plasmids* |  |  |
| pUCm | pUC19 derivative, lacking *lacZ* and mcs (amp) | This work |
| pSpo0MFla | pUCm derivative, *spo0M* (amp) | This work |
| pΔ0M | *spo0MD::kan* | This work |
| pSGGS | *spo0M-dsred (sp)* | This work |
| pT7-MAT-FLAG | pT7:MATtag:MCS:FLAGtag (amp) | Commercial |
| pT7-Spo0M-FLAG | *spo0M:FLAGtag (amp)* | This work |
