# Supplementary material for: Analysis of Spo0M function in Bacillus subtilis
Source: PLoS One. 2017 Feb 24;12(2):e0172737. doi: 10.1371/journal.pone.0172737 (PMC5325327; doi:10.1371/journal.pone.0172737)
Supplement: S3 Table — (DOCX) [file pone.0172737.s012.docx]

S3 Table

| Oligonucleotide | Sequence |
| --- | --- |
| pUCmFw | TTA ACT GCA GAA CCA ATG CAT TGT ACA TTC AAA TAT GTA TCC GCT CA |
| pUCmRv | AAA GGG ATC CCG AGC CGG AAG CAT AAA GTG TAA AG |
| SpoFlaFw | TAA ACT GCA GAA CAA TGC ATT GCT AAA AAA ACA AAG GAG G |
| SpoFlaRv | TAT GGG ATC CCG CTC GGC ACC AAT TTT AC |
| SpoXIFw | AAT CCG CTC GAG CAA CGA CTC CTT TAT TTT CAA TAT TAT T |
| SpoKIIRv | AAT GGG GTA CCC GTC TAG GAT CTC TTC TAG CAC |
